# Broad Electrocardiogram Syndromes Spectrum: From Common Emergencies to Particular Electrical Heart Disorders—Part II

**DOI:** 10.3390/diagnostics15121568

**Published:** 2025-06-19

**Authors:** Alexandr Ceasovschih, Anastasia Balta, Victorița Șorodoc, Krishnaraj Rathod, Ahmed El Gohary, Serghei Covantsev, Richárd Masszi, Yusuf Ziya Şener, Alexandru Corlăteanu, Syed Haseeb Raza Naqvi, Alexandra Grejdieru, Nicholas G. Kounis, Laurențiu Șorodoc

**Affiliations:** 1 Faculty of Medicine, ‘Grigore T. Popa’ University of Medicine and Pharmacy, 700115 Iasi, Romania; alexandr.ceasovschih@yahoo.com (A.C.);; 2 2nd Internal Medicine Clinic, ‘Sf. Spiridon’ Clinical Emergency Hospital, 700111 Iasi, Romania; 3 Centre for Cardiovascular Disease and Devices, William Harvey Research Institute, Queen Mary University of London, London E1 4NS, UK; k.s.rathod@qmul.ac.uk; 4 Interventional Cardiology Department, St. Bartholomew’s Hospital, London EC1A 7BE, UK; 5 King Fahad Specialist Hospital, Tabuk 47717, Saudi Arabia; aabdelwahabelgohary@yahoo.com; 6 Department of Surgical Oncology, Federal State Autonomous Institution National Medical Research Centre “Treatment and Rehabilitation Centre” of the Ministry of Health of the Russian Federation, 115478 Moscow, Russia; kovantsev.s.d@gmail.com; 7 Heart and Vascular Center, Semmelweis University, 1122 Budapest, Hungary; masszi.richard@gmail.com; 8 Thoraxcenter, Department of Cardiology, Erasmus MC University Medical Center, 3015 GD Rotterdam, The Netherlands; yzsener@yahoo.com.tr; 9 Department of Pulmonology and Allergology, “Nicolae Testemitanu” State University of Medicine and Pharmacy, 2004 Chisinau, Moldova; alexandru.corlateanu@usmf.md; 10 Ch Pervaiz Elahi Institute of Cardiology, Multan 60000, Pakistan; haseebraza51214@yahoo.com; 11 Department of Cardiology, “Nicolae Testemitanu” State University of Medicine and Pharmacy, 2004 Chisinau, Moldova; alexandra.grejdieru@usmf.md; 12 Department of Internal Medicine, Division of Cardiology, University of Patras Medical School, 26334 Patras, Greece; ngkounis@otenet.gr

**Keywords:** Long QT syndrome, Jervell and Lange–Nielsen syndrome, Romano–Ward syndrome, Andersen-Tawil syndrome, Timothy syndrome, Short QT syndrome, Twiddler’s syndrome, Noonan syndrome, Barlow’s Syndrome, Bundgaard syndrome, Danon disease, BRASH syndrome, Naxos disease, Carvajal syndrome

## Abstract

The electrocardiogram (ECG) remains a cornerstone of modern cardiology, providing rapid, non-invasive, and widely accessible diagnostic insights. While ECG interpretation is an essential skill for clinicians, certain patterns can be subtle or atypical, posing diagnostic challenges. In our previous review (doi.org/10.3390/jpm12111754), we explored several uncommon ECG syndromes with significant clinical implications. However, the spectrum of electrocardiographic abnormalities extends far beyond those initially discussed. In this second installment, we expand our discussion of rare and underrecognized ECG syndromes, including Long QT, Jervell and Lange-Nielsen, Romano–Ward, Andersen–Tawil, Timothy, Short QT, and Twiddler’s syndromes, as well as Noonan, Barlow’s, Bundgaard, BRASH, Carvajal, Naxos, and Danon disease. We highlight their clinical context, characteristic findings, and implications for diagnosis and management. These conditions range from acute, life-threatening emergencies requiring immediate intervention to chronic electrical disorders necessitating long-term monitoring and risk stratification. By broadening our focus, we aim to enhance awareness and recognition of these entities, ultimately improving patient outcomes through timely and accurate diagnosis.

## 1. Introduction

The electrocardiogram (ECG) remains a fundamental tool in cardiology, tracing its evolution from Einthoven’s early work to contemporary applications in clinical settings and wearable technologies [1]. In our previous review, “Broad Electrocardiogram Syndromes Spectrum: From Common Emergencies to Particular Electrical Heart Disorders” [2], we highlighted several syndromes characterized by distinct ECG patterns, aiding in their timely recognition and management. However, the spectrum of ECG syndromes is vast, and numerous conditions warrant further discussion. Given the diversity and clinical significance of these syndromes, we felt the need to expand our synthesis and explore additional entities that may challenge even experienced clinicians. Many of these syndromes are associated with distinct physical characteristics that, if recognized, can prompt clinicians to investigate ECG anomalies, just as an abnormal ECG pattern may lead to the identification of an underlying genetic or structural cardiac disorder. Crucially, some of these conditions predispose seemingly healthy young individuals to sudden cardiac death (SCD), underscoring the importance of early detection, risk stratification, and timely intervention. This second installment continues our deep dive into rare and underrecognized ECG patterns, emphasizing their pathophysiology, clinical manifestations, ECG diagnosis (Table 1), alternative diagnostic modalities, differential diagnoses, and guiding principles for patient care. By broadening our scope, we aim to provide clinicians with a more comprehensive understanding of these electrical heart disorders and their clinical implications, equipping them with a valuable resource for recognition and clinical approach.

## 2. Long QT Syndrome

Long QT syndrome (LQTS) is a genetically heterogeneous cardiac channelopathy characterized by prolonged ventricular repolarization, manifesting as a prolongation of QT interval (QTc). The prevalence of congenital LQTS is estimated to range from approximately 1 in 2000 to 1 in 2500 individuals in the general population [3]. The first description of LQTS dates back to 1956, when German pediatrician Meissner reported the case of a deaf student who suffered SCD due to emotional stress while being scolded by a teacher [4]. Shortly thereafter, in 1957, Jervell and Lange-Nielsen described a family presenting with congenital deafness, prolonged QTc, and SCD. This condition was later classified as Jervell and Lange-Nielsen syndrome (JLNS), an autosomal recessive form of LQTS, caused by homozygous or compound heterozygous mutations in the *KCNQ1* or *KCNE1* genes, which encode subunits of the IKs potassium channel [5]. In 1963 and 1964, Romano and Ward independently described families with a prolonged QTc and syncope but without deafness. This autosomal-dominant form, now termed Romano–Ward syndrome, is more common than JLNS [6]. Research conducted in the 1970s and 1980s further elucidated the clinical features and familial nature of LQTS, leading to the recognition of its genetic heterogeneity [7]. *KCNQ1* (LQT1), *KCNH2* (LQT2), and *SCN5A* (LQT3) were the first three LQTS-associated genes to be identified, marking a significant advancement in the 1990s. To date, at least seven genes have been conclusively proven to cause LQTS, with strong evidence linking mutations in these genes to the disorder. Additionally, numerous other genes have been implicated, with at least 17 genes currently being associated with the syndrome [4,8].

*Pathophysiology*. The prolonged QTc in LQTS is primarily caused by mutations in genes encoding cardiac ion channels. Approximately 95% of these mutations involve loss-of-function mutations in potassium channels. Specifically, the *KCNQ1* and *KCNE1* genes (LQTS1) are associated with the slow delayed rectifier potassium currents (IKs), while *KCNH2* and *KCNE2* (LQTS2) affect the rapid delayed rectifier potassium currents (IKr). Interestingly, the LQTS3 phenotype results from a gain-of-function mutation in *SCN5A*, leading to incomplete inactivation of the sodium channels (*I*Na) [7]. These alterations lead to an extended action potential duration in cardiac myocytes. The resultant prolonged repolarization predisposes individuals to malignant ventricular arrhythmias, particularly torsades de pointes (TdP) [9].

*Clinical manifestations*. The clinical manifestations of LQTS are diverse, varying according to the genetic subtype. A study by Rohatgi et al. involving 606 LQTS patients reported that only 27% were symptomatic, with the average age of symptom onset being 12 years [10]. The most common clinical manifestation is syncope, occurring in approximately 60% of untreated patients [11]. A detailed medical history is critical for diagnosis, as syncope in LQTS is distinct: it is not preceded by presyncope, palpitations, or orthostatic symptoms. Additionally, episodes may be associated with apnea, cyanosis, and tonic–clonic movements. There have been reports of overdiagnosis in LQTS, highlighting the importance of a careful and thorough diagnostic approach. This includes obtaining a detailed personal and family history, with attention to seizures, episodes of syncope, the circumstances surrounding syncope, potential precipitating triggers, and any family history of sudden death, drownings, or unexplained car accidents [7,12,13].

**Figure 1:**
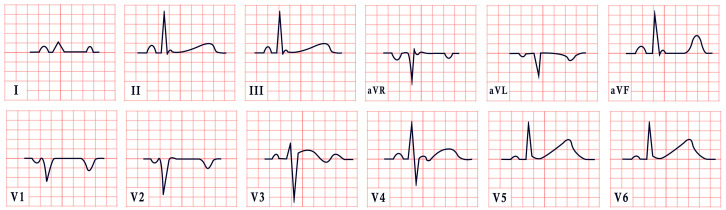
Electrocardiogram aspect in Long QT syndrome.

*ECG diagnosis.* It is essential to exclude acquired causes of QT prolongation and to perform serial ECGs to ensure diagnostic accuracy [13]. The hallmark ECG feature of LQTS is a prolonged QTc, reflecting delayed ventricular repolarization. Accurate QT interval measurement is crucial. On a standard 12-lead ECG, the QT interval should be measured manually, especially when the T wave is notched or flat, from the onset of the QRS complex to the end of the T wave, excluding U waves, typically in leads II, V2, or V3. The value should be averaged over at least three consecutive beats during stable sinus rhythm. Since the QT interval shortens with higher heart rates, correction formulas are used. Bazett’s formula is commonly applied between 60 and 100 bpm but may overestimate or underestimate QTc at heart rate extremes; the Framingham or Fridericia formulas may provide more accurate values [14]. In selected cases, 24 h Holter monitoring may be used to assess dynamic changes in QTc, particularly during sleep or recovery phases. However, QT measurement on Holter should be performed only during stable, artifact-free sinus rhythm, and is not reliable in the presence of frequent premature contractions, atrial fibrillation, or pacing. Abnormal QTc findings on Holter should always be confirmed with a standard 12-lead ECG [15]. A key step in the diagnostic process is ruling out drug-induced QT prolongation, which remains one of the most frequent acquired causes. High-risk agents include class Ia and III antiarrhythmics (e.g., quinidine, sotalol, amiodarone), antipsychotics (e.g., haloperidol, thioridazine), antimalarials (e.g., artemether/lumefantrine), and oncologic agents (e.g., arsenic trioxide, ribociclib, vandetanib) [16]. According to the 2022 ESC guidelines, such medications are contraindicated in patients with congenital LQTS and should be avoided if QTc >500 ms or increases >60 ms from baseline. A baseline ECG is recommended before initiating these agents, followed by repeat ECGs after a day and one to two weeks after starting or titrating the dose [17]. Additional ECG findings suggestive of LQTS include inverted T waves, typically observed in leads aVR and V1–V3, as well as T-wave notching and T-wave alternans (TWAs) (Figure 1). TWAs are a significant warning sign associated with lethal arrhythmias and SCD and should alert clinicians to heightened risk. Sinus pauses may also be present, serving as an additional alarming sign, particularly in cases of LQT3 [18,19]. Although rare, patients may also suffer from ventricular arrhythmias, atrioventricular blocks, and sinus bradycardia [20]. The most common ventricular arrhythmia in LQTS is TdP. TdP is typically pause-dependent, often triggered by a sinus pause following a short–long–short sequence. It is characterized by a rapid ventricular rate (160–250 bpm) and polymorphic QRS complexes that vary in morphology and axis, creating the characteristic “twisting of the points” appearance around the isoelectric line [21].

*Paraclinical diagnosis.* Ambulatory ECG monitoring is not considered the gold standard, but it may be useful for detecting transient QT prolongation or T-wave abnormalities, particularly at rest, when the LQT3 variant is suspected. Implantable loop recorders can provide long-term monitoring and aid in risk stratification [22,23]. Additional diagnostic tools, such as adrenaline challenge tests, mental stress tests, and supine-to-standing ECGs, may be employed, though they are generally regarded as having lower diagnostic value. Genetic testing plays a crucial role in confirming an LQTS diagnosis and identifying specific subtypes. It is strongly recommended (Class I) for patients with a Schwartz score of 3.5 or higher, asymptomatic individuals with a QTc >480 ms in early childhood or >500 ms in adolescence on at least two separate ECGs, and relatives of individuals with a confirmed disease-causing variant [13].

*Diagnostic criteria*. According to the Expert Consensus Statement on Inherited Arrhythmias, LQTS can be diagnosed in the following cases: (1) a Schwartz score ≥ 3.5 without any secondary causes, (2) a QTc ≥ 500 ms on repeated ECGs, or (3) a QTc of 480–499 ms accompanied by unexplained syncope [24]. The Schwartz score assigns points to clinical signs, ECG features, and family history to estimate LQTS probability. Scores range from 0 to 9, with ≥ 3.5 indicating a high LQTS probability. The clinical features included in the Schwartz score are syncope (1–2 points), congenital deafness (0.5 points), and a family history of LQTS or unexplained sudden death (0.5–1 points). The ECG features included are a QTc interval ≥ 480 ms (3 points), 460–479 ms (2 points), or 450–459 ms (1 point); TWA (1 point); notched T waves in three leads (1 point); and bradycardia (0.5 points) [12,25]. A modified version of the Schwartz score, endorsed by the 2022 European Society of Cardiology (ESC) guidelines on ventricular arrhythmias and sudden cardiac death, introduces additional diagnostic elements, including QTc ≥ 480 ms during the fourth minute of stress test recovery (1 point), and the presence of a causative mutation (3.5 points), while assigning greater weight to a resting QTc ≥ 480 ms (3.5 points) [17].

*Treatment*. Effective LQTS management relies on accurate risk stratification and appropriate lifestyle modifications to reduce the risk of life-threatening arrhythmic events. According to the current ESC guidelines, an invasive electrophysiological study is not routinely recommended in LQTS, but may be employed in select cases to aid differential diagnosis [17]. Instead, risk stratification and treatment decisions are based on clinical history, ECG findings, and genetic information to identify the patients at the highest risk of SCD who may require more intensive interventions [26]. According to the 2020 ESC guidelines on sports cardiology, individuals with the LQT2 subtype should avoid sports involving cold-water exposure. Additionally, all symptomatic patients are advised to refrain from competitive sports. Asymptomatic individuals with confirmed pathogenic mutations should receive counseling and engage in a shared decision-making process to guide their participation in physical activities [27]. Beta-blockers are the mainstay of LQTS therapy, reducing the cardiac event risk by up to 60% and recommended for all diagnosed patients. Potassium channel openers (e.g., nicorandil, pinacidil) are considered for LQT2, while mexiletine and ranolazine may be used in beta-blocker-intolerant or refractory cases. Magnesium and potassium supplements serve as adjuncts, especially for recurrent arrhythmias. ICDs are indicated for LQTS patients with prior cardiac arrest or recurrent syncope despite optimal therapy, with implantation decisions based on the SCD risk assessment [28]. Pacemakers may help patients with bradycardia-induced QT prolongation, while left cardiac sympathetic denervation can reduce arrhythmias in beta-blocker-intolerant or refractory cases. Catheter ablation is an emerging option for recurrent arrhythmic events despite medical and device therapy [26].

## 3. Jervell and Lange-Nielsen Syndrome

Jervell and Lange-Nielsen syndrome (JLNS) is a rare autosomal recessive disorder characterized by the co-occurrence of congenital sensorineural hearing loss and a prolonged QTc interval. It has an estimated prevalence of approximately 1 in 1000,000 individuals [11,25]. The condition arises from homozygous or compound heterozygous mutations in the *KCNQ1* or *KCNE1* genes, inherited from both parents. These mutations cause a more severe LQTS phenotype. Individuals with JLNS face a markedly elevated risk of life-threatening arrhythmias, such as TdP, which frequently manifest during early childhood [13].

*Pathophysiology*. The hallmark of JLNS is a disrupted slow delayed rectifier potassium current (IKs), which prolongs the cardiac action potential and QTc on ECG. This defect arises from biallelic mutations in *KCNQ1* or *KCNE1*, affecting both cardiac and cochlear cells. Impaired ion channel function in the inner ear causes sensorineural hearing loss by disrupting potassium cycling within the cochlea, essential for auditory signal transmission [11,29].

*Clinical manifestations*. In a study conducted by Schwartz et al., 86% of individuals with JLNS were symptomatic, with 15% developing symptoms within the first year of life. The vast majority experienced a major cardiac event before the age of 18. The most common triggers for these events were physical exercise and emotional stress, with swimming identified as a particularly frequent precipitant. Only 7% of events occurred during rest or sleep. A smaller proportion of patients reported events associated with gestation, pyrexia, sepsis, diarrhea, or hypokalemia [30]. Profound bilateral sensorineural hearing loss, congenital in nature, is often identified through newborn screening programs or audiometric testing. Cardiac symptoms—syncope, palpitations, and cardiac arrest—are common and often triggered by physical exercise or emotional stress. Without appropriate intervention, the risk of mortality exceeds 50% by adolescence due to life-threatening ventricular arrhythmias [31].

**Figure 2:**
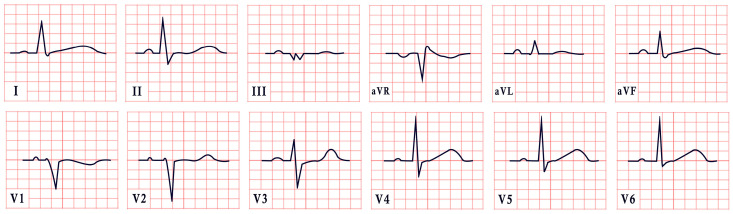
Electrocardiogram aspect in Jervell and Lange-Nielsen syndrome.

*ECG diagnosis.* Key electrocardiographic findings include a markedly prolonged QTc, with a QTc typically exceeding 500 ms, which is significantly longer than that observed in autosomal-dominant forms of LQTS. T waves may present as broad, notched, or biphasic, with variations depending on the underlying genetic mutation (Figure 2). Additionally, bradycardia is often observed, resulting from the extended duration of ventricular repolarization [32].

*Paraclinical diagnosis.* Audiometric testing consistently confirms profound bilateral sensorineural hearing loss, a defining feature of JLNS. Genetic testing serves as a definitive diagnostic tool, identifying pathogenic variants in the *KCNQ1* or *KCNE1* genes [32].

*Diagnostic criteria.* JLNS diagnosis is established based on

1. Prolonged QTc > 500 ms.
2. Congenital bilateral sensorineural hearing loss.
3. Family history of consanguinity or SCD.
4. Genetic confirmation of mutations in *KCNQ1* or *KCNE1* [25,33].

The Schwartz score for LQTS diagnosis is of limited utility in cases of congenital deafness [33].

*Differential diagnosis.* Romano–Ward syndrome, Alport syndrome, Usher syndrome, acquired LQTS.

*Treatment*. Treatment for LQTS aims on reducing arrhythmic risk and improving quality of life. Beta-blockers such as propranolol and nadolol are the first-line therapy, reducing arrhythmogenic triggers. ICDs are recommended for high-risk patients, particularly those with a history of cardiac arrest or recurrent syncope. In patients with JLNS, cochlear implants help to restore auditory function and improve communication. Lifestyle modifications, including avoiding QT-prolonging drugs and stressful stimuli, are essential [34,35]. While prognosis remains guarded without treatment, timely interventions significantly reduce mortality [32,36].

## 4. Romano–Ward Syndrome

Romano–Ward syndrome (RWS) is an autosomal-dominant form of LQTS that includes subtypes LQT1-6 and LQT9-16. It is characterized by prolonged ventricular repolarization in the absence of extracardiac manifestations, such as hearing loss, skeletal anomalies, or neurodevelopmental abnormalities. RWS has a global prevalence of approximately 1 in 2500 individuals and demonstrates significant variability in clinical presentation and symptom severity among those affected [32].

*Pathophysiology*. Mutations in up to 15 genes encoding cardiac ion channel subunits or regulatory proteins have been implicated in RWS, with the most frequent mutations occurring in *KCNQ1* (LQT1), *KCNH2* (LQT2), and *SCN5A* (LQT3) [37]. These genetic mutations prolong the action potential duration and delay cardiac repolarization, predisposing individuals to early afterdepolarization. This mechanism increases the risk of life-threatening arrhythmias, particularly during periods of physical or emotional stress [38].

*Clinical manifestations*. According to the phenotype, there may be different precipitants for cardiac events. In LQT1, the most common precipitants are strong emotions and physical exertion, particularly activities such as running or swimming. In contrast, LQT2 shares these triggers but also includes auditory stimuli such as alarm clocks, sirens, or a phone ringing. Notably, approximately 40% of LQT2 patients may experience cardiac events during rest or sleep. For LQT3, the majority of cardiac events occur during sleep [19]. Aborted cardiac arrest or SCD may be the initial manifestation in up to 10% of untreated patients [11]. The age at which SCD occurs differs by phenotype: in LQT1, it is most frequently observed in late childhood and early adolescence; in LQT2, it typically arises during mid-to-late adolescence or early adulthood; and in LQT3, it generally occurs between late adolescence and the third decade of life [7].

**Figure 3:**
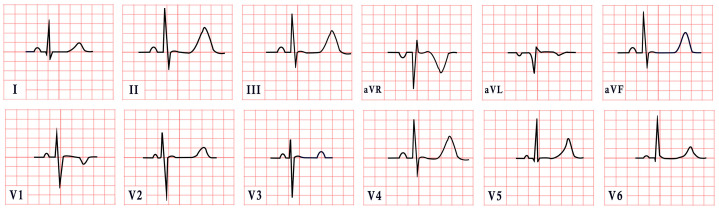
Electrocardiogram aspect in Romano–Ward syndrome.

*ECG diagnosis*. In a study by Priori et al., the mean corrected QTc was 466 ± 44 ms for LQT1, 490 ± 49 ms for LQT2, and 496 ± 49 ms for LQT3 [38]. However, it is important to note that up to 25% of patients with genetically confirmed LQTS may present with a normal QTc interval. T-wave morphology varies depending on the specific genetic mutation. In LQT1, T waves may appear normal, broad-based, asymmetric, or display a late onset with a peaked “infantile” shape. In LQT2, they are often of a lower amplitude and may exhibit notching. In LQT3, T waves can be delayed in onset, appear distant from the QRS complex, or demonstrate an asymmetric contour (Figure 3).

*Paraclinical diagnosis.* A diagnostic approach similar to that used for LQTS can be employed for the diagnosis of RWS [32].

*Diagnostic criteria*. The Schwartz score is considered the gold standard for initial risk stratification; a high score may indicate the need for genetic testing, which remains the definitive method for diagnosis, enabling the identification of specific ion channel mutations associated with the syndrome [39].

*Differential diagnosis*. Differential diagnosis can be performed by distinguishing prolonged QTc caused by inherited conditions, such as other subtypes of LQTS and Brugada syndrome, from those with acquired causes, including drug-induced QT prolongation. It is essential to evaluate and exclude other contributing factors, such as electrolyte disturbances, hypothyroidism, and ischemic heart disease, which can mimic the ECG findings of LQTS [40].

*Treatment*. Management strategies prioritize arrhythmia prevention. Beta-blockers, particularly propranolol or nadolol, are commonly prescribed to reduce the incidence of cardiac events by lowering heart rate and sympathetic activity. For patients at high risk of SCD, an ICD may be considered. Avoiding QT-prolonging drugs and sudden stressors is also critical. With proactive management, prognosis is generally favorable, though regular follow-up and lifestyle adaptations are essential for high-risk patients [32].

## 5. Andersen–Tawil Syndrome

Andersen–Tawil syndrome (ATS), also referred to as Andersen Cardiodysrhythmic Periodic Paralysis, is a rare autosomal-dominant channelopathy characterized by a triad of periodic paralysis, ventricular arrhythmias, and distinctive facial or skeletal dysmorphisms. Historically classified as LQTS type 7 (LQT7), ATS has an estimated prevalence of 1 in 1000,000. The syndrome was first identified in 1963 by Klein, a pediatrician in Boston, Massachusetts [41,42,43]. In 1971, Danish physician Ellen Andersen expanded upon the initial observations by describing the classic symptom triad. In 1994, neurologist Rabi Tawil significantly advanced understanding of the disorder [44,45].

*Pathophysiology*. ATS has an autosomal-dominant inheritance pattern and includes at least two proposed subtypes based on genetic findings. Andersen–Tawil syndrome type 1 (ATS1), which accounts for approximately 60% of cases, is caused by mutations in the *KCNJ2* gene, with over 21 distinct mutations identified. This gene encodes the Kir2.1 inward rectifier potassium channel, which is crucial for maintaining the resting membrane potential and phase 3 repolarization in cardiac myocytes. Mutations in *KCNJ2* reduce IK1 current, prolonging repolarization and predisposing to ventricular arrhythmias. In contrast, Andersen–Tawil type 2 (ATS2), comprising about 40% of cases, refers to patients who lack mutations in *KCNJ2*. Although mutations in *KCNJ5* have been proposed in some cases, these findings are not widely accepted, and the genetic basis of ATS2 remains uncertain [46]. The electrophysiological hallmark of ATS is impaired potassium ion flow, leading to delayed repolarization. However, unlike classical LQTS, QTc prolongation in ATS may be mild, intermittent, or even absent [47].

*Clinical manifestations.* ATS is classically characterized by a triad of features: arrhythmias, periodic paralysis, and physical anomalies. In terms of cardiovascular manifestations, common symptoms include palpitations and syncope, while no specific triggers have been identified. Notably, SCD has been reported in approximately 50% of patients. Periodic paralysis typically presents at a very young age, often preceding the onset of cardiovascular symptoms by approximately two years. This paralysis and accompanying muscular weakness is not directly dependent on potassium levels, although it occurs more frequently in patients with hypokalemia. The duration of these episodes is variable, sometimes lasting up to several days [48,49]. Craniofacial anomalies associated with ATS include low-set ears, wide-set eyes, and a hypoplastic mandible. In a few cases, a long, narrow head shape, named scaphocephaly, has been reported, likely resulting from premature sagittal suture fusion. Skeletal abnormalities commonly observed include clinodactyly of the fifth digit, syndactyly of the second and third toes, and scoliosis. However, not all individuals with ATS exhibit the full spectrum of these features [49,50]. Neuropsychiatric associations (e.g., attention deficit hyperactivity disorder, depression) have been suggested by some authors but remain unconfirmed [51,52].

**Figure 4:**
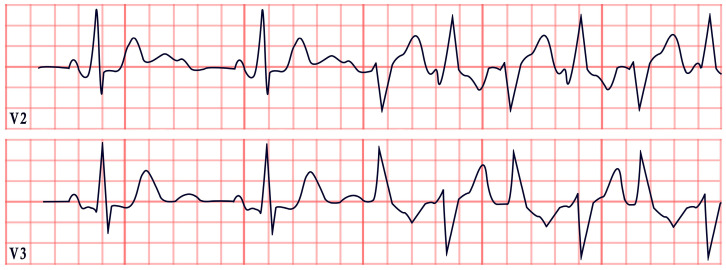
Electrocardiogram aspect in Andersen–Tawil syndrome.

*ECG diagnosis.* Studies have identified several cardiovascular abnormalities associated with ATS. These include prominent U waves in leads V2–V4, bidirectional ventricular tachycardia, and frequent premature ventricular contractions. There is ongoing debate regarding QTc duration. Earlier studies included the U wave in the measurement, suggesting a prolonged QT interval. However, many recent authors argue that QTc remains within the normal range, while the Q-U interval is prolonged, averaging 655 msec compared to 600 msec in controls (Figure 4) [53]. Importantly, arrhythmias in ATS can occur independent of marked QT prolongation. In a study by Barron-Diaz et al., 50% of patients demonstrated polymorphic ventricular tachycardia on Holter monitoring, underscoring the arrhythmogenic potential of the syndrome regardless of the QTc duration [43,48].

*Paraclinical diagnosis.* Additional investigations that may support diagnosis include Holter ECG monitoring to detect arrhythmias, measurement of blood potassium levels at baseline and during symptomatic episodes, and thyroid function assessment. In a study by Vivekandam et al., 65% of ATS patients had a normal echocardiogram, while the remainder exhibited either diastolic or systolic dysfunction. Electromyography, muscle MRI, and biopsy may help to differentiate ATS from other neuromuscular disorders [54]. Genetic testing can identify specific mutations in affected individuals, aiding in diagnosis confirmation and family screening [55].

*Diagnostic criteria*. Several diagnostic criteria have been proposed, with a diagnosis of ATS considered when at least two of the following four criteria are met:

1. Periodic paralysis.
2. Cardiac manifestations such as ventricular arrhythmias (e.g., frequent ventricular ectopic beats or ventricular tachycardia), a prolonged QTc, and/or a prominent U wave.
3. Dysmorphic features, with at least two of the following: low-set ears, wide-set eyes, a small mandible, fifth-digit clinodactyly, or syndactyly.
4. A family history of confirmed ATS [55].

*Differential diagnosis.* The differential diagnosis for a prolonged QTc includes other forms of LQTS such as RWS, JLNS, and Timothy syndrome. ATS can also occur in catecholaminergic polymorphic ventricular tachycardia.

*Treatment*. As a genetic condition, ATS currently has no curative treatment, and management focuses on symptom control. Arrhythmias, periodic paralysis, and syncope are typically addressed with pharmacotherapy or implantable devices. Beta-blockers, calcium channel blockers, or flecainide may be used, while patients with a history of cardiac arrest or sustained ventricular tachycardia have an indication for an implantable cardioverter defibrillator [17,42]. Periodic paralysis can be treated with carbonic anhydrase inhibitors like acetazolamide, and potassium supplementation may help correct hypokalemia in some cases. Medications that prolong the QTc should be avoided to minimize risks. While competitive or strenuous sports should be discouraged due to the arrhythmia risk, light physical activity is encouraged, as it may reduce paralysis severity [49,54].

## 6. Timothy Syndrome

Timothy syndrome (TS) or Long QT syndrome type 8 is a rare channelopathy with multi-organ involvement, including congenital heart defects, facial malformations, syndactyly, developmental delay, and immunodeficiencies [56]. The first reported case was described by Reichenbach et al. in 1992 at the University of Leipzig, Germany [57]. TS was later illustrated in detail in 2004 by Splawski and Timothy from the Universities of Massachusetts and Utah, respectively. The syndrome was named after Katherine Timothy in recognition of her numerous years of dedicated research on the disorder [58].

*Pathophysiology*. TS follows an autosomal-dominant inheritance pattern, though most reported cases result from de novo mutations [59]. The disorder arises from mutations in the *CACNA1C* gene, which encodes the Cav1.2 L-type calcium channel, crucial for calcium influx in cardiac, neuronal, and other excitable tissues. Due to the pathogenic mutations channel inactivation is impaired, leading to prolonged calcium influx, which leads to markedly prolonged ventricular action potentials and QTc intervals [60]. Two types of TS have been described: TS1 results from a mutation in exon 8A, while TS2 is caused by a mutation in exon 8 [61]. Since exon 8 is expressed approximately four-times more frequently in cardiac and neural tissues than exon 8A, some authors suggest that TS2 has a more severe prognosis [62].

*Clinical manifestations*. Patients with TS are often born prematurely due to fetal bradycardia and are highly vulnerable to general anesthesia, with multiple reports of cardiac arrest during procedures [63]. Syncope was also reported in approximately 70% of patients [60]. TS1 is characterized by distinct craniofacial features, including a receding hairline, rounded facial structure, underdeveloped jaw, flattened nasal bridge, thin upper lip, and a predisposition to dental caries. In contrast, TS2 exhibits greater variability in physical traits, with fewer documented cases in the literature; however, a receding hairline, flattened nasal bridge, and thin upper lip appear to be common findings [61]. Syndactyly is unique to the classical TS1 phenotype [63]. Neurologic manifestations include speech delay, impaired communication, and ataxia. Immune dysfunction with recurrent infections and hypoglycemia has also been reported [58].

**Figure 5:**
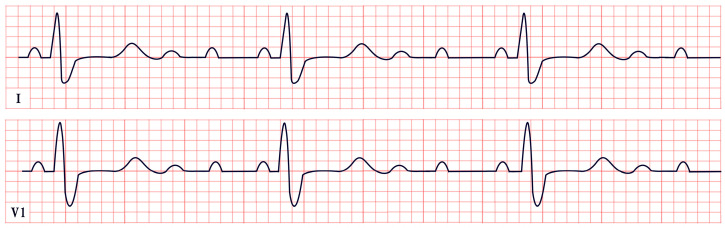
Electrocardiogram aspect in Timothy syndrome.

*ECG diagnosis.* All TS patients exhibit prolonged QT intervals, with a QTc exceeding 480 ms in TS1 and sometimes surpassing 600 ms in TS2 [61]. In a study by Dufendach et al., 47% of patients had a 2:1 atrioventricular block, while 6% experienced bradycardia due to sinus pauses. Additionally, 47% displayed T-wave alternans (Figure 5) [56]. Life-threatening arrhythmias, including ventricular tachycardia, ventricular fibrillation, and cardiac arrest, have also been reported (Figure 5) [64].

*Paraclinical diagnosis.* Echocardiographic findings in TS patients are primarily derived from case reports, as no large studies exist due to the rarity of the syndrome and the high mortality rates. These reports describe structural abnormalities such as patent ductus arteriosus, patent foramen ovale, ventricular septal defects, and tetralogy of Fallot. Left ventricular hypertrophy has also been noted, although a case reported by Hiippala et al. documented a structurally normal heart [61,62,63,64]. Genetic testing for the specific mutation should be conducted [59].

*Diagnostic criteria*. Although there is no established consensus on the diagnostic criteria for TS, diagnosis is typically based on

1. Characteristic craniofacial features (e.g., receding hairline, flat nasal bridge, thin upper lip) and, in TS1, syndactyly.
2. ECG findings, particularly QTc prolongation (>480 ms in TS1, often > 600 ms in TS2), and 2:1 AV block, history of lethal ventricular arrhythmias, or cardiac arrest.
3. Identification of a pathogenic mutation in the *CACNA1C* gene (exon 8A for TS1, exon 8 for TS2) [56,59].

*Differential diagnosis*. Andersen–Tawil syndrome, Jervell and Lange-Nielsen syndrome, Long QT syndrome.

*Treatment*. There are no established data regarding the efficacy of medical therapy in TS. Beta-blockers have been used but should be administered cautiously, with careful monitoring of glycemic levels due to their potential to mask hypoglycemia [65]. Other suggested antiarrhythmic treatments include calcium channel blockers, mexiletine, and ranolazine to reduce the arrhythmogenic risk. Surgical correction is advised for structural heart defects [59]. Due to the high mortality associated with TS, ICD implantation is considered essential, even in asymptomatic patients. Additionally, pacemaker implantation is recommended for those with sinus pauses and significant bradycardia [63].

## 7. Short QT Syndrome

Short QT syndrome (SQTS) is a rare, genetically determined, autosomal-dominant, cardiac channelopathy characterized by a markedly shortened QTc on ECG and a heightened risk of life-threatening arrhythmias and SCD. The first report linking a shortened QTc to an elevated risk of SCD was published by Algra et al. in 1993 in Rotterdam, the Netherlands [66]. In 2000, the genetic underpinnings of the condition were further elucidated with the discovery of mutations associated with its pathophysiology by Ihor Gussak in Rochester, Minnesota [67].

*Pathophysiology*. SQTS arises from genetic mutations that disrupt cardiac ion channel function, resulting in abnormally accelerated repolarization of the cardiac action potential. Subtypes SQTS 1–3 are associated with gain-of-function mutations in potassium channels, which increase delayed rectifier potassium currents (IKr or IKs), thereby shortening the action potential [68]. In contrast, SQTS 4-6 involve loss-of-function mutations in calcium channels, resulting in reduced calcium currents (ICa), also contributing to action potential abbreviation. Additionally, a novel SQTS subtype has been linked to mutations in the cardiac chloride/bicarbonate exchanger AE3, possibly affecting membrane repolarization [69]. Consequently, SQTS is characterized by a shortened QTc, diminished atrial and ventricular effective refractory periods, and increased transmural dispersion of repolarization, which creates a substrate for reentrant arrhythmias. The syndrome is genotypically and phenotypically heterogeneous, with seven subtypes identified to date, each linked to mutations in specific cardiac ion channel genes [70].

*Clinical manifestations*. Approximately 40% of patients with SQTS are asymptomatic. Among symptomatic individuals, cardiac arrest is the most common initial presentation, occurring in 28% of cases, and the most frequent symptom overall, with a prevalence of 34% [71]. SCD occurs in approximately 4% of patients during the first year of life and shows two incidence peaks: one in infancy and another between the third and fifth decades of life [72]. Other notable manifestations include palpitations, dizziness, and syncope [73]. The triggers for cardiac events may vary by SQTS subtype. For instance, in SQTS1 patients, exertion and emotional stress are common precipitants, whereas SQTS3 has been associated with nocturnal palpitations. Notably, a systematic review of 40 studies revealed that the incidence of cardiac events and SCD in SQTS patients is significantly higher among women [74].

**Figure 6:**
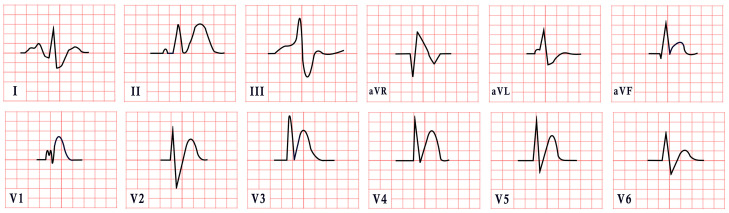
Electrocardiogram aspect in Short QT syndrome.

*ECG diagnosis*. SQTS is characterized not only by a shortened QTc but also by its failure to appropriately adapt to heart rate changes. The QTc remains relatively constant, with inadequate prolongation during bradycardia and excessive shortening during tachycardia: a phenomenon known as pseudonormalization. This distinctive pattern can aid in diagnosis, particularly when confirmed through serial ECGs, Holter monitoring, or treadmill testing. These modalities are especially useful in patients with elevated baseline heart rates or sinus bradycardia. For optimal accuracy, experts recommend obtaining ECGs at a heart rate of approximately 60 bpm [72,75]. These tools also address potential misdiagnoses due to Bazett’s formula, which may overcorrect the QTc at slower heart rates. Additional ECG findings in SQTS include the shortening or absence of the ST segment, tall and peaked or biphasic T waves, and prominent U waves (Figure 6). Atrial fibrillation, premature ventricular contractions, ventricular fibrillation, and cardiac arrest may occur [75,76].

*Paraclinical diagnosis*. Treadmill stress testing may reveal a QTc that remains unchanged or shows minimal adaptation to heart rate variations, with a QT/HR slope < 0.9 ms/beat/min [76]. Speckle-tracking echocardiography and tissue Doppler imaging may reveal impaired systolic activity [72]. The role of electrophysiological studies in SQTS diagnosis remains controversial. Some studies report markedly brief atrial and ventricular refractory periods and increased susceptibility to induced atrial and ventricular fibrillation [73,75]. However, electrophysiological studies do not predict the risk of SCD [72]. Seven genetic mutations have been linked to SQTS, but the genotype–phenotype correlation remains unclear due to the limited number of genetically confirmed cases [73,75].

*Diagnostic criteria*. SQTS diagnosis employs the “Schwartz score for SQTS” proposed by Veltmann et al., combining ECG findings, clinical history, family history, and genetic testing. A QTc < 370 ms earns one point, < 350 ms earns two, and < 330 ms earns three, with one additional point for a J point–T peak interval < 120 ms. Clinical criteria include episodes of cardiac arrest or polymorphic VT/VF (two points each), syncope without secondary causes, or atrial fibrillation (one point each). Family history awards two points for relatives with likely SQTS and one point for cardiac arrest or cot death. Genetic findings add two points for a pathogenic mutation or one for other mutations. At least one ECG point is required in order for the other criteria to apply [77].

*Differential diagnosis*. Brugada syndrome, early repolarization syndrome.

*Treatment*. ICD therapy is the cornerstone of management, particularly for patients with clinical manifestations, a positive family history, or supportive findings on electrophysiological studies or genetic testing. Importantly, a negative genetic test or electrophysiological study does not exclude the diagnosis or eliminate the risk of future arrhythmic events [73]. Pharmacological therapy may be considered in certain situations for patients with contraindications to ICD implantation or who decline the procedure, or as an adjunctive therapy to minimize appropriate ICD discharges and manage symptomatic atrial fibrillation episodes. Quinidine is considered the most effective treatment for SQTS, as it inhibits multiple potassium currents and blocks inward sodium and calcium currents, thereby prolonging the QTc and ventricular refractory periods [78,79,80]. Disopyramide, particularly effective in SQTS1, also extends the QTc and ventricular refractory periods, making it a viable alternative to quinidine [81]. Other antiarrhythmic agents have generally demonstrated limited or no efficacy in SQTS. Flecainide was initially thought to slightly prolong the QTc and reduce ventricular fibrillation inducibility, but its utility remains unclear. Propafenone has shown effectiveness in managing frequent paroxysms of AF without affecting the QTc, with no arrhythmia recurrence reported for over two years [82]. However, IKr blockers such as ibutilide and sotalol have been ineffective for SQTS patients [78].

## 8. Twiddler’s Syndrome

Twiddler’s syndrome, first described by Bayliss in 1968, results from pacemaker malfunction due to the patient’s handling of the pacemaker leads, either consciously or unconsciously. This condition occurs when rotation of the pulse generator under the skin causes coiling and malpositioning of the leads, typically within the first 12 months following implantation. The prevalence of Twiddler’s syndrome has been estimated at approximately 1.2% in a high-volume U.S. center based on real-world data [83,84].

*Pathophysiology*. In a review by Gomez et al., patients receiving antidepressants were more prone to manipulate the pacemaker. While many patients may adjust or move the device to check its position or alleviate pain, most cease this behavior over time. However, psychiatric patients tend to persist in this behavior long after implantation [84]. Another important risk factor is the discrepancy between the size of the pacemaker pocket and the pacemaker itself, which can be especially evident in obese patients [85].

*Clinical manifestations*. Skin abrasions may be evident at the level of manipulation [86]. Lead displacement can cause various symptoms, most frequently phrenic nerve stimulation, which may result in hiccups or diaphragmatic contractions, along with sensations of abdominal pulsations and rhythmic arm twitching when the brachial plexus is stimulated [87]. Pacemaker dysfunction may lead to fatal arrhythmias and cardiac arrest [86].

**Figure 7:**
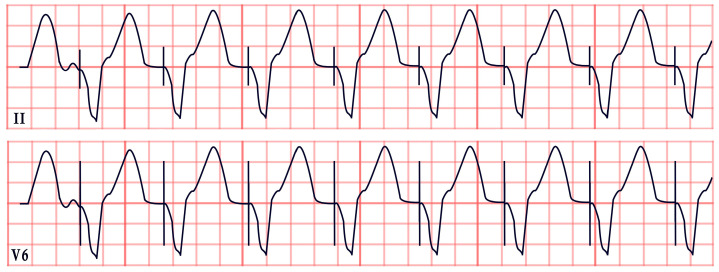
Electrocardiogram aspect in Twiddler’s syndrome.

*ECG diagnosis*. ECG findings vary depending on the pacemaker’s indication, type, and programming. They may range from normal readings to complete heart block with loss of capture. Notable signs include the absence of pulse-generator artifacts, loss of capture, inappropriate pacing, abnormal pacing morphology, irregular pacing intervals, and asynchronous pacing. In cases involving ICDs, loss of adequate sensing and capture can result in more dramatic ECG findings, including malignant ventricular arrhythmias and asystole (Figure 7) [88].

*Paraclinical diagnosis*. ECG and chest X-ray are the main pillars of the diagnosis. Chest X-ray typically reveals displacement of the lead from its original position, often appearing coiled near the generator. Additionally, pacemaker interrogation can identify dysfunction [88].

*Differential diagnosis*. Reel syndrome—a syndrome resulting from inappropriate anchoring of the leads during implantation. The generator spins around its long axis in the pocket.

*Treatment*. The treatment involves reoperation and repositioning of the lead securely by applying additional sutures to anchor the generator tightly to the fascia within the surgical pocket. Patient education and, in some cases, a psychiatric evaluation may be necessary to prevent recurrence [85,88].

## 9. Noonan Syndrome

Noonan syndrome (NS) is an autosomal-dominant genetic disorder with an estimated incidence of 1 in 1000–2500 live births, affecting multiple organ systems [89]. The syndrome is characterized by short stature, distinctive facial features, developmental delay, and cardiovascular abnormalities, including congenital heart defects [90]. Initially considered a “male Turner syndrome”, the condition was redefined in 1963 by Noonan and Ehmke, who identified it as a distinct disorder in patients exhibiting Turner-like phenotypic features without the chromosomal abnormalities of Turner syndrome [91,92].

*Pathophysiology*. NS is an autosomal-dominant disorder, although de novo mutations have also been reported [93]. NS arises from a disruption of the RAS/MAPK signaling pathway, which is normally triggered by cytokines, hormones, and growth factors. This pathway is essential for both early and late developmental processes, including the regulation of organ development, synaptic adaptability, and cellular growth. NS is caused by mutations in several genes, with 80–85% of cases attributed to *PTPN11*, *SOS1*, *RAF1*, *RIT1*, *KRAS*, *NRAS*, *MRAS*, *BRAF*, *LZTR1*, *SOS2*, *RASA2*, *RRAS*, and *MAP2K1* [94,95,96,97].

*Clinical manifestations*. Hearing abnormalities are present in approximately 25% of patients, primarily affecting high frequencies, while 10% experience impairments in low frequencies [89]. Congenital heart disease affects up to 90% of NS patients [92]. The most common heart defects encountered in this condition are pulmonary valve stenosis (25–75%) and atrial septal defects (4–57%). However, the list of abnormalities includes ventricular septal defects (1–14%), atrioventricular canal defects (1–13%), mitral valve defects (2–17%), aortic coarctation (2–9%), and patent ductus arteriosus (1–4%) [97,98]. Craniofacial features evolve with age: infancy is marked by a prominent forehead, low-set ears, and short neck; toddlers may develop macrocephaly, broad nasal bridge, and hypertelorism; childhood features often appear reduced or inexpressive; and adolescence brings an inverted triangular face with a pointed chin, broad forehead, and webbed neck [92]. Hair is typically fine and sparse during early childhood, often becoming curly with advancing age. Eye color is distinctively blue or green and various opthalmological abnormalities may be present. Infants with NS may exhibit feeding difficulties, episodes of vomiting, diarrhea, and steatorrhea, and delays in developmental milestones. Cryptorchidism is frequently observed in males, while females generally maintain normal fertility. Renal agenesis, hypoplasia, distal ureteric stenosis, and other abnormalities may occur, but are commonly rare. Some patients may have subtle bruising or clinically evident splenomegaly [89,92].

**Figure 8:**
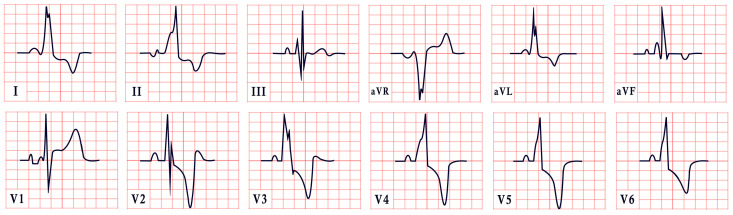
Electrocardiogram aspect in Noonan syndrome.

*ECG diagnosis*. Patients with NS often exhibit distinctive ECG features, including left-axis deviation with a negative aVF, an abnormal R/S ratio over the left precordium, and abnormal Q waves (Figure 8) [99]. These findings were historically attributed to factors such as counterclockwise rotation of the heart, structural cardiac defects, and conduction abnormalities [100,101]. However, a study by Raaijmakers et al. involving 118 patients with NS demonstrated that 58% exhibited a characteristic ECG pattern. Importantly, the study found no statistically significant relationship between the presence of these ECG abnormalities and underlying structural cardiac defects, suggesting that the distinctive ECG features in NS may arise independently of structural heart abnormalities [99]. ECG abnormalities are unequally distributed across age groups, with a higher prevalence observed in infants and young children [102].

*Paraclinical diagnosis*. Echocardiography plays a crucial role in diagnosing Noonan syndrome, revealing left ventricular hypertrophy (LVH) and frequently associated anomalies such as pulmonary valve stenosis and mitral valve dysplasia [103]. According to a review by Allanson et al., excessive LV posterior wall thickening, symmetric hypertrophy, and a septal-to-posterior wall ratio <2 are markers of a poor prognosis. A comprehensive blood panel is necessary to assess thyroid function, insulin-like growth factor-1 (IGF-1), and coagulation factors, given the increased risk of nutritional deficiencies and hematological disorders. Abdominal ultrasound is recommended, as ~50% of patients develop splenomegaly due to underlying hematologic or oncologic conditions. Renal ultrasound should be performed, as 11% of individuals with Noonan syndrome present with renal abnormalities. Regular ophthalmologic evaluations are crucial due to the high prevalence of ocular abnormalities. Genetic testing is essential for differentiating Noonan syndrome from other syndromes with overlapping phenotypic features [92].

*Diagnostic criteria*. van der Burgt et al. established a diagnostic scoring system with major and minor criteria. Major criteria include characteristic facial dysmorphism, cardiac defects such as pulmonary valve stenosis or HOCM, stature below the third percentile, chest deformities, a family history of NS, and features including intellectual disability, cryptorchidism, and lymphatic abnormalities. Minor criteria encompass suggestive facies, other cardiac abnormalities, height below the 10th percentile, a broad chest, and mild developmental delays. Diagnosis is confirmed with a characteristic facial dysmorphism and either one major or two minor criteria, or suggestive facial features with two major or three minor criteria [104].

*Differential diagnosis*. Differential diagnosis can be made with Turner syndrome, fetal alcohol syndrome, Mosaic trisomy 22, 8p duplication syndrome, Williams–Beuren syndrome, Baraitser–Winter cerebrofrontofacial syndrome, Aarskog–Scott syndrome, and Costello syndrome.

*Treatment*. Management involves a comprehensive approach across various age stages, including screening of multiple organ systems, monitoring growth and development, and providing targeted treatments and interventions when necessary [104].

## 10. Barlow’s Disease

Barlow’s disease (BD) is a clinical syndrome that was once considered benign but is now recognized as a significant cause of malignant arrhythmias and SCD. While its prevalence in the general population is 2–3%, BD predominantly affects women, who also have a higher risk of SCD, with a reported prevalence of up to 13% [105]. Initially, its characteristic non-systolic ejection click had been attributed to pleuropericardial adhesions. However, in 1965, John Brereton Barlow et al. challenged this notion in a landmark article describing seven clinical cases. His findings, which linked the condition to mitral valve prolapse, were met with controversy at first, as they contradicted the prevailing understanding of the syndrome. Over time, Barlow’s work became widely accepted, reshaping the clinical perspective on this disease [106,107].

*Pathophysiology*. BD is characterized by the superior displacement of the mitral leaflet by more than 2 mm during systole. It results from fibro-myxomatous changes in the mitral valve tissue, leading to prolapse into the left atrium [108,109]. When assessing the risk of SCD in BD, a study by Basso et al. found that all SCD victims with BD had fibrosis in the left ventricular papillary muscles, and 88% also had fibrosis at the inferior base of the heart. This myocardial fibrosis is believed to contribute to the arrhythmogenic potential of BD, even when patients are hemodynamically stable [110]. The most common arrhythmic presentation associated with mitral valve prolapse (MVP) is premature ventricular complexes, followed by nonsustained ventricular tachycardia (VT), sustained VT, polymorphic VT, and ventricular fibrillation [105,111]. A key mechanism behind PVCs is stretch-induced depolarization, which may either present as an isolated finding or act as a trigger for more severe arrhythmias [112]. Additional contributing factors include fibrotic changes, excessive traction on the papillary muscles, increased sympathetic activity, and decreased vagal tone, all of which promote ectopic ventricular activity [113].

*Clinical manifestations*. Patients with BD commonly experience chest pain, palpitations, exertional breathlessness, syncope, and hypotension. On auscultation, BD is characterized by a late systolic murmur and a non-ejection systolic click [114]. In a systematic review of SCD cases in mitral valve prolapse patients by Han HC et al., 58% of patients experienced palpitations before cardiac arrest, 28% were asymptomatic, and in 47% of cases, SCD was precipitated by physical or emotional stress [115].

**Figure 9:**
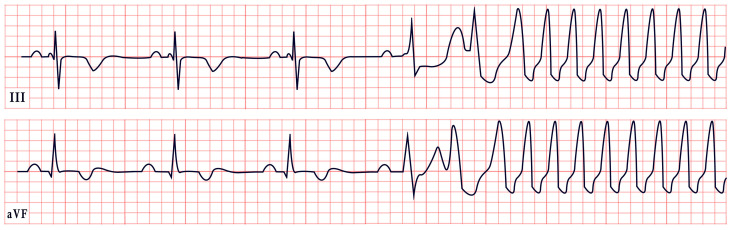
Electrocardiogram aspect in Barlow’s syndrome.

*ECG diagnosis*. ECG abnormalities are observed in 58–89% of patients with mitral valve prolapse [116]. In a review by Han HC et al. on the electrical activity of SCD patients with Barlow’s syndrome, 51% exhibited premature ventricular complexes, 24% had inverted T waves, and approximately 32% had a normal ECG (Figure 9). On 24 h Holter monitoring, PVCs and couplets were found in 63% of patients, while nonsustained ventricular tachycardia was present in 29% [115].

*Paraclinical diagnosis*. Cardiac imaging plays a crucial role in diagnosing mitral valve prolapse and assessing arrhythmic risk. Transthoracic echocardiography and Doppler ultrasound are used to evaluate the severity of regurgitation, while transesophageal echocardiography is recommended when valve visualization is suboptimal. Patients with a higher burden of ventricular arrhythmias (VAs) more frequently exhibit bileaflet mitral valve prolapse and mitral annular disjunction. Another proposed echocardiographic marker for VA risk is increased anterior mitral leaflet length during systole, along with the Pickelhaube sign, which is a Doppler tissue signal, greater than 16 cm/s, resembling a spiked helmet. Cardiac magnetic resonance (CMR) further refines risk stratification, with late gadolinium enhancement in the papillary muscles and the inferior and basal LV strongly associated with arrhythmogenicity, as it indicates macroscopic fibrosis. Additionally, shortened T1 mapping times, suggesting widespread microscopic fibrosis, have emerged as another marker of arrhythmic risk in these patients [105].

*Diagnostic criteria*. A set of macroscopic and microscopic histological criteria have been proposed.

Macroscopic criteria:

1. Ballooning or hooding of the mitral leaflets between the chordae tendineae.
2. Arching of the leaflets into the left atrium.
3. Enlargement of the leaflets.
4. Increase in the mitral valve area due to dilation of the annulus.

Microscopic criteria:

1. Enlargement of the spongiosa and fibrosa layers.
2. Deposition of fibrin and platelets.
3. Changes in the atrialis layer, where the collagen fibers become compacted [Guy TS., 2012].

*Differential diagnosis*. Fibroelastic deficiency.

*Treatment*. Arrhythmia management in MVP remains challenging, as ventricular arrhythmias often persist despite valve repair [117]. Catheter ablation is recommended for symptomatic or high-risk VT, while severe mitral regurgitation necessitates surgical intervention [111]. Some studies suggest that mortality owing to ventricular arrhythmias decreases after MV surgery when mitral annular disjunction resolves, though the occurrence of major arrhythmic events remains unpredictable [118]. Following surgical MV repair or replacement, an ICD is a Class I recommendation for patients who meet ESC guidelines for the secondary prevention of SCD [32]. The annual incidence of SCD in MVP is estimated at 0.2–0.4%, increasing to 1.8% in cases with severe MR due to leaflet flail, underscoring the need for lifelong follow-up in high-risk patients [111].

## 11. Danon Disease

Danon disease (DD) is a metabolic disorder characterized by cardiac, skeletal muscle, and neurological involvement. First described by Moris Danon in 1981, it was identified in two 16-year-old boys presenting with cardiomegaly, myopathy, and cognitive impairment. Muscle biopsies revealed excessive glycogen accumulation in skeletal muscle, leading to its initial classification as a lysosomal storage disease [119,120]. In 2000, Ichizo Nishino et al. identified the underlying cause as a mutation in a lysosomal membrane protein (LAMP2), rather than a lysosomal enzyme, clarifying the pathophysiology of the disorder [121].

*Pathophysiology*. DD follows an X-linked dominant inheritance pattern, though some cases of de novo mutations have been reported. Causative mutations occur in the *LAMP2* gene, located on the X chromosome. This gene encodes three protein isoforms involved in lysosomal protein degradation [122]. All known pathogenic mutations affect the *LAMP*-*2B* isoform, which is predominantly expressed in tissues such as the heart, skeletal muscle, and brain [123].

*Clinical manifestations*. Symptoms manifest earlier and progress more rapidly in male patients. Approximately 90% of affected males develop proximal muscle weakness, which gradually worsens, though it is not the primary cause of death. In contrast, myopathy appears to affect only 35–50% of females [123,124]. In a study by Boucek et al., 88% of males and 77% of females with DD were symptomatic, with palpitations being the most common symptom, followed by chest pain [125]. Patients may also experience breathlessness, exercise intolerance, and fatigue. SCD has been reported. Mild cognitive impairment is observed in up to 70% of patients [122]. Ophthalmological manifestations, including scotomas, blurry vision, and reduced visual acuity, have also been described [126]. Additionally, more than half of patients, particularly males, report respiratory and gastrointestinal symptoms [125].

**Figure 10:**
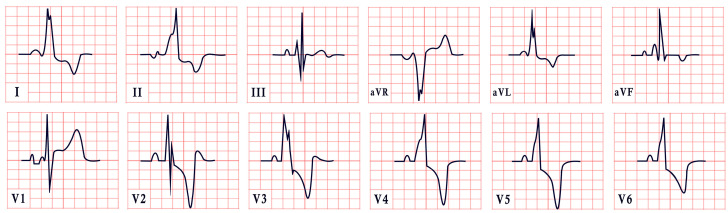
Electrocardiogram aspect in Danon disease.

*ECG diagnosis*. ECG abnormalities are present even before patients develop symptoms, serving as an early indicator of the disease [127]. In a study by Lotan et al., 50% of males and 41% of females exhibited the characteristic shortened PR interval and delta wave of Wolff–Parkinson–White (WPW) syndrome on ECG [119]. WPW is a common finding in metabolic disorders, including Pompe disease [127]. ECG may also reveal abnormal Q waves, atrioventricular blocks, atrial fibrillation, inverted T waves, and, notably, pronounced signs of left ventricular hypertrophy, which are often evident even in the early stages of the disease, particularly in males (Figure 10). Severe arrhythmias, such as ventricular fibrillation, sustained ventricular tachycardia, and cardiac arrest, have also been reported [119,128,129].

*Paraclinical diagnosis*. In a study by Cheng et al., DD patients exhibited biomarker elevations, including creatine kinase and liver function enzymes, at levels three times the normal compared to controls [128]. Echocardiography typically reveals left ventricular hypertrophy with a preserved ejection fraction, characteristic of concentric cardiomyopathy. Over time, this progresses to dilated cardiomyopathy [124]. Interestingly, females may have asymmetric left ventricular hypertrophy, and both hypertrophic and dilated cardiomyopathies [130]. CMR is valuable for assessing the fibrosis extent and distinguishing DD from other cardiomyopathies [124,127]. In DD, LGE is diffuse, and the interventricular septum is usually spared [130]. Electromyography, along with ophthalmologic assessments such as Humphrey visual field testing and electroretinography, should be performed [126,130]. Genetic testing is also recommended [130].

*Diagnostic criteria*. Hong et al. proposed a set of diagnostic criteria, distinguishing between men and women.

For women, diagnosis requires a confirmed LAMP2 mutation and, at least one cardiovascular sign, such as

1. ECG abnormalities.
2. Late gadolinium enhancement on CMR.
3. Left ventricular hypertrophy.
4. Ejection fraction < 50%.

For men, diagnosis requires a confirmed LAMP2 mutation, and either one cardiovascular sign (as listed above), or at least one of the following:

1. Muscular abnormalities, detected by EMG, or elevated biomarkers, such as liver enzymes or creatine kinase at two times the normal limit.
2. Neurocognitive signs, such as attention deficit, or developmental delay [130].

*Differential diagnosis*. Pompe disease, Fabry disease, familial hypertrophic cardiomyopathy, Friedreich ataxia, Duchenne muscular dystrophy.

*Treatment*. Currently, there are no specific guidelines for treating DD. However, heart failure and arrhythmias should be managed according to existing guidelines. ICD placement is recommended for symptomatic patients with nonsustained ventricular tachycardia and syncope. Catheter ablation may be performed for WPW [128]. Heart transplantation is an option, though some studies report poor outcomes in males, with death occurring within a year post-transplant [119,130].

## 12. BRASH Syndrome

BRASH syndrome (bradycardia, renal failure, atrioventricular nodal blockade, shock, and hyperkalemia) describes a clinical condition where the synergistic effects of AV nodal blockers and acute kidney injury create a cycle of severe bradycardia and hyperkalemia. A constellation of these symptoms was first described by Thomas Lee et al. in 1986 at Harvard Medical School, Boston, Massachusetts [131]. Nevertheless, BRASH syndrome as an entity has only recently been recognized, and it remains relatively new, understudied, and often underdiagnosed, as it is frequently mistaken for a simple electrolyte imbalance [132,133]. BRASH syndrome proves lethal in 5.7% of cases [134].

*Pathophysiology*. The pathophysiology of BRASH syndrome involves an initial trigger, such as dehydration or drug up-titration, leading to acute kidney injury. This results in impaired potassium excretion and hyperkalemia, which exacerbates bradycardia and reduces cardiac output, further worsening renal function and perpetuating the cycle [135]. In a systematic review by Shah et al., only 40% of BRASH patients had chronic kidney disease, suggesting that pre-existing renal dysfunction is not a prerequisite. In total, 83% of cases were precipitated by beta-blockers, 45% by calcium channel blockers, and 23% by ACE inhibitors or ARBs [133]. Dehydration-induced hypovolemia has also been identified as a common trigger, particularly during hot months [136].

*Clinical manifestations*. In a systematic review by Majeed et al., the most frequently reported symptoms were related to cerebral hypoperfusion, including syncope, lightheadedness, drowsiness, and encephalopathy. Additionally, 17% of patients experienced gastrointestinal symptoms, including nausea, vomiting, or abdominal discomfort, while approximately 13% reported breathlessness. Chest pain was a less common presentation, occurring in around 5% of cases, but may have been underreported due to the patients’ altered general status [134].

**Figure 11:**
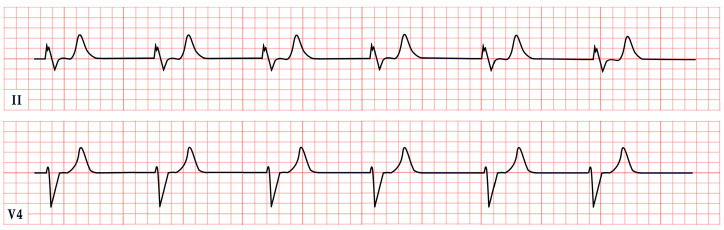
Electrocardiogram aspect in BRASH syndrome.

*ECG diagnosis*. Hyperkalemia alone can induce bradycardia, but this typically occurs when potassium levels exceed 7.0 mEq/L. ECG changes due to hyperkalemia, such as peaked T waves, usually appear at levels of 5.5–6.0 mEq/L and progressively worsen, with QRS widening and bradycardia occurring at more severe elevations (>7.0 mEq/L) [137]. However, in BRASH syndrome, the expected ECG changes of hyperkalemia are often absent. Bradycardia in BRASH syndrome is commonly associated with junctional escape rhythm (50%), sinus bradycardia (17.1%), and complete heart block (12.9%) (Figure 11). Atrial fibrillation or atrial flutter with a slow ventricular response may be present in approximately 10% of cases [134].

*Paraclinical diagnosis*. Laboratory markers are assessed to evaluate electrolyte imbalances and renal function. In a systematic review of the literature, the median potassium level was 6.3 mEq/L, blood urea nitrogen was 73 mg/dL, serum creatinine was 2.8 mg/dL, serum bicarbonate was 15 mEq/L, and lactate was 7.8 mmol/L [133].

*Differential diagnosis*. Isolated hyperkalemia, intoxication with AV-node blocking medication.

*Treatment*. Treatment of BRASH syndrome includes fluid resuscitation, correction of hyperkalemia (using intravenous calcium, insulin/glucose, beta-agonists, and diuretics), and management of bradycardia, which may necessitate epinephrine infusion. Additional therapies such as glucagon and atropine have been suggested, but their effectiveness is questionable. In a systematic review of 34 studies, atropine and glucagon were largely ineffective in reversing symptoms, and 59.5% of patients required inotropic or chronotropic support [133,135].

## 13. Bundgaard Syndrome

Bundgaard syndrome, also known as “Familial ST-segment depression syndrome”, is an autosomal-dominant inherited cardiac arrhythmia first identified by Bundgaard Henning et al. in 2018 [138]. The condition was documented in five families from three different countries through collaborative research conducted at tertiary centers in Copenhagen, Amsterdam, and Oxford [138].

*Pathophysiology*. Bundgaard syndrome exhibits an autosomal-dominant inheritance pattern with high penetrance, although no causative gene has yet been identified. Histopathological examinations of affected individuals have revealed no consistent structural abnormalities, aside from nonspecific fibrosis and findings occasionally observed in cardiomyopathies [139]. Recent studies have sought to characterize the electrophysiological basis of the syndrome. In a study by Frosted et al., CineECG was used to assess differences in cardiac repolarization between individuals with Bundgaard syndrome and healthy controls. The researchers identified altered repolarization patterns localized to the base of the left ventricle. By experimentally reducing the action potential duration and amplitude in the basal myocardial areas of healthy individuals, they were able to replicate the ST-segment changes observed in affected patients, suggesting a functional basis for the electrocardiographic phenotype [140].

*Clinical manifestations*. ECG manifestations typically emerge during early adolescence; however, patients generally remain asymptomatic until approximately 50 years of age. They are predisposed to arrhythmias and commonly present symptoms such as palpitations, syncope, cardiac arrest, or SCD [139].

**Figure 12:**
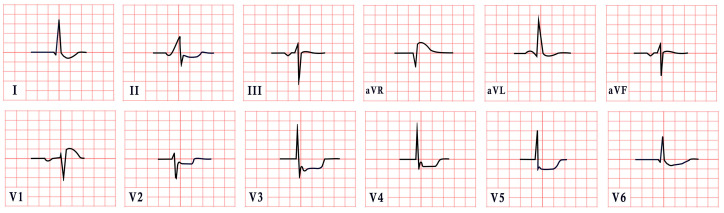
Electrocardiogram aspect in Bundgaard syndrome.

*ECG diagnosis*. In familial ST-depression syndrome, ST-segment depression is consistent and non-ischemic, showing no reaction to external factors or improvement due to known influences. Instead, the depression typically worsens during physical activity. It is most significantly seen in leads II, V5, and V6. In a study by Christensen et al., 13% of those studied experienced atrial fibrillation, while another 13% had a history of aborted cardiac arrest or SCD. Ventricular arrhythmias were noted without any concurrent alterations in the QTc, ST-segment, or T-wave morphology, and they were mainly of a polymorphic nature (Figure 12) [139,141].

*Paraclinical diagnosis*. Cardiac stress testing may reveal persistent ST-segment depression. Holter ECG proves beneficial for evaluating the arrhythmic burden and risk assessment. In a study by Christensen et al., the majority of patients preserved a normal ejection fraction (EF); however, the EF was notably lower in men, with a quarter of patients displaying an EF under 50%. Notably, CMR reveals no specific structural abnormalities [139,142].

*Diagnostic criteria*. The following criteria have been proposed:

1. Concave and up-sloping ST-segment depression greater than 0.1 mV in at least four leads (V3–V6, I–III), measured 80 milliseconds after the J-point, in the absence of a secondary cause.
2. ST-segment elevation greater than 0.1 mV observed in the lead aVR.
3. Persistent ST-segment abnormalities, with no evidence of normalization over time.
4. ST-segment depression exacerbated with exertion.
5. Evidence of autosomal-dominant inheritance [139].

*Differential diagnosis*. Brugada syndrome, LQTS, left main coronary disease.

*Treatment*. The approach to treating Bundgaard syndrome is mainly symptomatic, concentrating on addressing the patient’s clinical symptoms. ICD implantation might be considered for patients who face recurrent syncopal episodes or are at high risk for severe arrhythmias [141].

## 14. Naxos Disease

Naxos disease is a rare autosomal recessive disorder characterized by a combination of cardiac and cutaneous manifestations. It was first described by Nikos Protonotarios et al. in 1986, in four families from the Greek Cycladic island of Naxos, with an estimated prevalence of approximately 0,01% [143,144,145].

*Pathophysiology*. Naxos disease is an autosomal recessive disorder caused by a deletion in the junction plakoglobin (*JUP*) gene on chromosome 17 [146]. *JUP* encodes plakoglobin, a key component of desmosomes and adherens junctions, which facilitates cell–cell adhesion in cardiac and epithelial tissues. It interacts with desmin filaments and the actin cytoskeleton. Mutations lead to the apoptosis of cardiomyocytes, and fibrofatty replacement, ultimately contributing to arrhythmogenic cardiomyopathy [145]. The disease primarily affects the right ventricle with transmural fibrofatty replacement, though the left ventricle may also be involved, with various grades of severity [147].

*Clinical manifestations*. From early life, most patients present curly and woolly hair, which many authors have suggested as an indicator of poor cardiovascular prognosis. Eyebrows and eyelashes may also be pronounced [147]. Cutaneous lesions typically appear within the first two years of life, in the hands and legs, initially sparing the dorsal areas before gradually spreading diffusely. Their well-defined borders are an important aid in differential diagnosis [144]. Some patients are born with shorter limbs, along with curved nails, and dental anomalies such as hypoplastic or fewer teeth [145,148]. Syncope and sustained ventricular tachycardia are common clinical manifestations, with approximately 33% of patients developing symptoms before the age of 30. In some cases, SCD may be the initial presentation. The cardiomyopathy may progress gradually or follow a pattern of “hot phases”, sudden episodes of ventricular tachycardia, accompanied by structural deterioration, resembling acute myocarditis [147].

**Figure 13:**
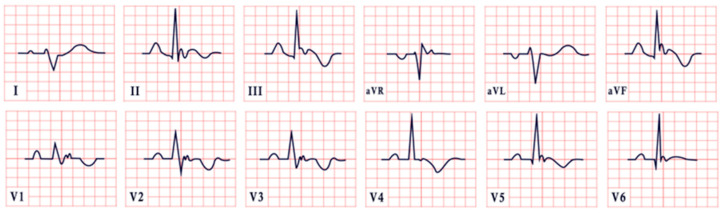
Electrocardiogram aspect in Naxos disease.

*ECG diagnosis*. ECG abnormalities are common in symptomatic patients, with approximately 90% of those with Naxos disease displaying changes. These include negative T waves in leads V1-V3 or V1-V6 and epsilon waves, which are small, low-amplitude positive deflections occurring after the QRS complex and before the onset of the T wave, present in 38% of cases (Figure 13) [147,149]. Ventricular tachycardia with a complete or LBBB pattern is frequent, while atrial fibrillation has been reported in a minority of cases [144].

*Paraclinical diagnosis*. Transthoracic ultrasound frequently reveals a dilated RV outflow tract, often accompanied by an RV aneurysm. Kinetic abnormalities, particularly in the lateral wall of the RV, are also commonly observed. CMR is used to assess the extent of fibrosis and evaluate the risk of life-threatening arrhythmias, thus guiding treatment decisions. Genetic testing is recommended to confirm the diagnosis in patients and their family members. Although myocardial biopsy is rarely performed today, it may be employed in cases of inconclusive diagnosis [144].

*Diagnostic criteria*. Considering that Naxos disease is a genetic form of arrhythmogenic right ventricular cardiomyopathy, the 2024 Revised International Task Force Criteria published by Corrado et al. are applicable for diagnosis.

The major criteria include

1. Regional right ventricular kinetic abnormalities with RV systolic dysfunction or global dilation.
2. Histological evidence of fibrofatty replacement in more than one myocardial sample.
3. Electrocardiographic findings, including
  - Inverted T waves in leads V1–V3, excluding cases with complete right bundle branch block (RBBB).
  - ST-segment or J-point elevation in V1–V3, with the presence of epsilon waves in leads V1–V3.
  - Ventricular arrhythmias with a non-inferior axis, including premature ventricular beats or ventricular tachycardia (VT) (sustained or nonsustained).
4. Genetic confirmation of a pathogenic variant in a patient, with either a family member meeting diagnostic criteria or postmortem confirmation on autopsy.

The minor criteria include

1. Isolated regional RV kinetic abnormalities.
2. Gadolinium enhancement in more than one area of the RV, confirmed in two different orthogonal views.
3. ECG modifications:
  - Inverted T waves in leads V1–V2 (without complete right bundle branch block) in patients older than 14.
  - ST-segment or J-point elevation.
  - Inverted T waves beyond V3 with complete right bundle branch block.
  - Inverted T waves beyond V3 in patients younger than 14 years.
  - Terminal activation duration of the QRS ≥ 55 ms, measured from the nadir of the S wave to the end of the QRS in leads V1, V2, or V3.
4. Ventricular arrhythmias, including
  - Premature ventricular beats or VT with an inferior axis.
  - History of aborted cardiac arrest due to VT or ventricular fibrillation.
5. Genetic and family history criteria, including
  - Identification of a likely pathogenic gene variant.
  - History of a family member with probable arrhythmogenic cardiomyopathy, or a family member who died before age 35.
  - Confirmed arrhythmogenic cardiomyopathy in a second-degree relative [148,149].

*Differential diagnosis*. Arrhythmogenic cardiomyopathies with right or biventricular involvement, Carvajal syndrome, acute myocarditis.

*Treatment*. Patients should avoid strenuous physical exertion. Beta-blockers and flecainide are commonly used for antiarrhythmic management, though amiodarone remains controversial. In cases of refractory ventricular tachycardia, catheter ablation may be considered. Heart failure treatment should follow the current European Society of Cardiology guidelines, and in end-stage disease, heart transplantation may be an option [144,148].

## 15. Carvajal Syndrome

Carvajal syndrome, a syndromic form of desmoplakin (DSP) cardiomyopathy, is a cardiocutaneous disorder closely resembling Naxos disease, though it was identified nearly a decade later in Indian and Ecuadorian populations. First described by Luis Carvajal-Huerta in 1998 at Luis Vernaza Hospital in Ecuador, this syndrome follows either an autosomal recessive or autosomal-dominant inheritance pattern and is associated with mutations in at least three known genes [150,151,152]. Its prevalence remains unknown [153]. Unlike Naxos disease, Carvajal syndrome primarily affects the left ventricle, with minimal or no right ventricular involvement, and carries a high risk of arrhythmias and SCD [151,154].

*Pathophysiology*. Carvajal syndrome has been primarily associated with mutations in the *DSP* gene, located on chromosome 6p24, which encodes the DSP protein. *DSP* serves as a crucial link between *JUP* or plakophilins and intermediate desmin filaments, ensuring structural integrity in cardiac and epithelial tissues [146,155]. Additionally, mutations in desmocollin-2 and plakophilin-2 have also been identified in some cases [150]. While the exact mechanism remains unknown, myocardial injury appears to precede the onset of arrhythmias and heart failure, supporting the hypothesis that left ventricular fibrosis occurs before its dilation [151].

*Clinical manifestations*. Patients with Carvajal syndrome present with woolly, friable hair from birth. By around two years of age, they develop palmoplantar keratoderma, which differs from Naxos disease by being striate [156]. Some reports also describe linear skin keratosis in flexural regions and follicular involvement on the legs, knees, elbows, and abdomen. Vesicles and bullae may appear on the torso, while other features can include nail clubbing, leukonychia, hypodontia, or oligodontia [153]. In a study by Di Lorenzo et al., more than half of the patients reported no cardiovascular symptoms. Among those who were symptomatic, palpitations were the most common complaint, occurring in nearly 4/5 patients, followed by chest pain, which affected 44% of patients. Additionally, 39% reported myocarditis-like chest pain [157]. In a study by Wang et al., the initial presentation was aborted cardiac arrest in 4% of Carvajal syndrome patients, while 10% experienced SCD as their first manifestation [154].

**Figure 14:**
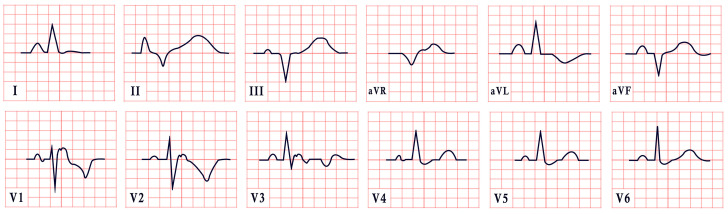
Electrocardiogram aspect in Carvajal syndrome.

*ECG diagnosis*. On ECG, 44% of patients exhibit a low QRS voltage in leads aVR, aVF, and aVL, while approximately 22% present with T-wave inversions in V2-V4 [151]. A study by López-Ayala et al. found that inverted T waves in V4-V6 had prognostic significance, aiding in early disease identification before structural abnormalities appeared (Figure 14) [158]. According to Cipriani et al., these low QRS amplitudes and T-wave inversions are characteristic of left ventricular cardiomyopathy and play a key role in differentiating it from dilated cardiomyopathy [159,160]. In a study by Di Lorenzo et al., 72% of patients exhibited polymorphic premature ventricular contractions [157]. Although uncommon, both left and right bundle branch blocks may occur [161].

*Paraclinical diagnosis*. Troponin levels rise during the “hot phases” of the disease. Echocardiography may not detect early-stage disease, as fibrosis is often confined to the epicardial layer, with reductions in the left ventricular ejection fraction and kinetic abnormalities appearing later in progression [151]. Coronary angiography is performed to rule out other causes of angina. CMR is the gold standard for diagnosis. A study by Augusto et al. demonstrated that Carvajal syndrome patients exhibit a characteristic ring-like late gadolinium enhancement (LGE) pattern, localized to the subepicardial layer [162]. Genetic testing for the DSP gene should be performed, as it is vital for the differential diagnosis of cardiomyopathies [163]. Endomyocardial biopsy is rarely performed and is most often conducted postmortem [157]. A study by Reza et al., showcased hypertrophic myocytes and fibrosis in the endocardium and interstitium in all examined subjects [164].

*Diagnostic criteria*. The 2024 Revised International Task Force Criteria published by Corrado et al. for arrhythmogenic cardiomyopathy of the left ventricular phenotype may be applied.

Major Criteria:

1. Characteristic ring-pattern late gadolinium enhancement (LGE) involving more than three left ventricular segments in at least two orthogonal views.
2. Low QRS amplitude in leads aVF, aVR, and aVL, excluding cases due to obesity, pulmonary emphysema, pericardial effusion, or cardiac amyloidosis.

Minor Criteria:

1. Global LV systolic dysfunction, which may occur without left ventricular dilation.
2. Regional late gadolinium enhancement involving the lateral, inferior, and/or septal wall segments in at least two orthogonal views.
3. T-wave inversions in leads V4–V6, excluding cases with complete left bundle branch block.
4. More than 500 premature ventricular contractions per 24 h with RBBB morphology, excluding ectopies arising from the His–Purkinje system.
5. Ventricular tachycardia (sustained or nonsustained) with an RBBB morphology, excluding ectopies arising from the His–Purkinje system.
6. Aborted cardiac arrest due to sustained ventricular tachycardia or ventricular fibrillation [149].

*Differential diagnosis*. Naxos disease, acute myocarditis, arrhythmogenic cardiomyopathy with LV or biventricular involvement.

*Treatment*. A study by Jacobsen et al. found that exercise exacerbates myocardial injury in Carvajal syndrome. While its exact impact on heart failure and arrhythmias remains unclear, endurance exercise should be avoided in affected patients and carriers [151,165]. Management of Carvajal syndrome includes arrhythmia control, heart failure treatment, and SCD prevention through ICD implantation [166].

## 16. Conclusions

In this review, we have outlined the characteristics, diagnostic modalities, and treatment principles of several rare and underrecognized ECG syndromes. By doing so, we hope to raise clinical suspicion and awareness, ultimately improving detection, appropriate risk stratification, and timely intervention. Recognizing these conditions is crucial, as many are associated with life-threatening arrhythmias and SCD, particularly in young and seemingly healthy individuals. Given the complexity of these syndromes, a multidisciplinary approach is often necessary for accurate diagnosis and optimal management. Awareness of these conditions is essential across multiple specialties to ensure timely recognition and referral. Additionally, continued research is needed to refine diagnostic criteria, improve risk stratification, and develop targeted therapeutic strategies. Through this review, we aim to contribute to a broader understanding of these syndromes and their clinical implications, equipping healthcare professionals with the knowledge to improve patient care. Raising awareness and encouraging further research will be key in advancing our ability to diagnose and manage these frequently underdiagnosed and undertreated conditions.

## Figures and Tables

**Table 1:** Key electrocardiographic findings in ECG syndromes.

ECG Syndrome	ECG Characteristics	ECG Aspect
Long QT Syndrome	-Prolonged QTc interval (>460 ms in females, >450 ms in males) -T-wave notching, inversion, and T-wave alternans	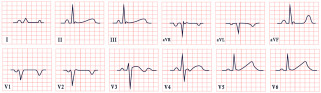
Jervell and Lange-Nielsen Syndrome	-QTc prolongation >500 ms -Broad, notched, or biphasic T waves	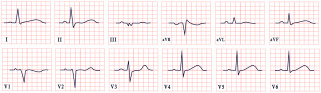
Romano–Ward Syndrome	-LQT1: QTc = 466 ± 44 ms, broad-based, asymmetric, or peaked ‘infantile’ T waves -LQT2: QTc = 490 ± 49 ms, low-amplitude T waves -LQT3: QTc = 496 ± 49 ms, remote or asymmetric T waves	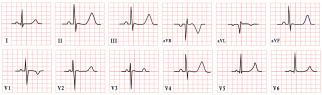
Andersen–Tawil Syndrome	-Prominent U waves in V2–V4 -Q-U prolongation >655 ms -QTc may be normal	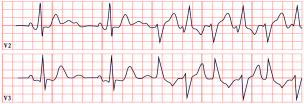
Timothy Syndrome	-QTc > 480 ms (TS1), >600 ms (TS2) -2:1 AV block -T-wave alternans	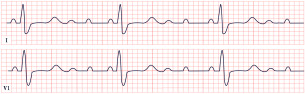
Short QT Syndrome	-Short QTc < 370 ms with poor HR adaptation -Short or absent ST segment -Tall, narrow, peaked, or biphasic T waves -Prominent U waves	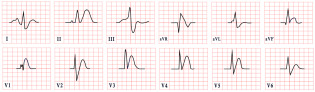
Twiddler’s Syndrome	-Loss of capture, abnormal pacing morphology -Irregular/asynchronous pacing -Asystole	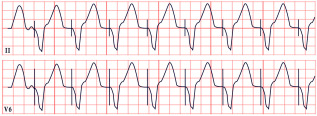
Noonan Syndrome	-Left axis deviation -Abnormal R/S ratio in V4–V6 -Abnormal Q waves	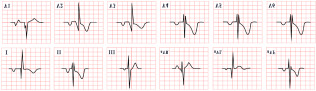
Barlow’s Syndrome	-Inverted T waves -PVCs	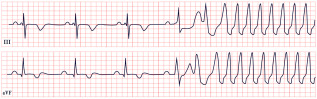
Danon Disease	-Short PR interval, Delta waves -Abnormal Q waves -AV block -Inverted T waves -Left ventricular hypertrophy	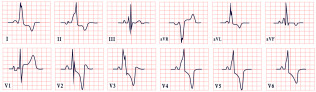
BRASH Syndrome	-Peaked T waves -Bradycardia, junctional rhythm	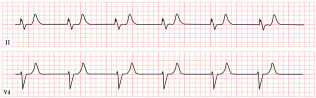
Bundgaard Syndrome	-Persistent ST depression in II, V5, V6 -Deepens with exertion, non-ischemic	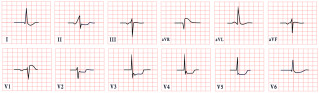
Naxos Disease	-Inverted T waves in V1–V3/V1–V6 -Epsilon waves	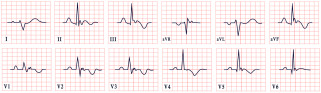
Carvajal Syndrome	-Low QRS voltage -Inverted T waves in V2–V6 -Epsilon waves	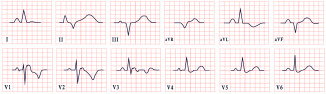

## References

[1] Friedman P.A. (2024). The electrocardiogram at 100 years: History and future. Circulation.

[2] Balta A., Ceasovschih A., Șorodoc V., Dimitriadis K., Güzel S., Lionte C., Stătescu C., Sascău R.A., Mantzouranis E., Sakalidis A. (2022). Broad electrocardiogram syndromes spectrum: From common emergencies to particular electrical heart disorders. J. Pers. Med..

[3] Viskin S. (1999). Long QT syndromes and torsade de pointes. Lancet.

[4] Li J. (2022). Long QT syndrome: Long story short. Eur. Heart J..

[5] Huang L., Bitner-Glindzicz M., Tranebjærg L., Tinker A. (2001). A spectrum of functional effects for disease causing mutations in the Jervell and Lange-Nielsen syndrome. Cardiovasc. Res..

[6] Romano C. (1963). Rare cardiac arrythmias of the pediatric age. II. Syncopal attacks due to paroxysmal ventricular fibrillation. (Presentation of 1st case in Italian pediatric literature). Clin. Pediatr..

[7] Vincent G.M. (2002). The long QT syndrome. Indian Pacing Electrophysiol. J..

[8] Adler A., Novelli V., Amin A.S., Abiusi E., Care M., Nannenberg E.A., Feilotter H., Amenta S., Mazza D., Bikker H. (2020). An international, multicentered, evidence-based reappraisal of genes reported to cause congenital long QT syndrome. Circulation.

[9] Shimizu W. (2005). The long QT syndrome: Therapeutic implications of a genetic diagnosis. Cardiovasc. Res..

[10] Rohatgi R.K., Sugrue A., Bos J.M., Cannon B.C., Asirvatham S.J., Moir C., Owen H.J., Bos K.M., Kruisselbrink T., Ackerman M.J. (2017). Contemporary outcomes in patients with long QT syndrome. J. Am. Coll. Cardiol..

[11] Goldenberg I., Moss A.J., Peterson D.R., McNitt S., Zareba W., Andrews M.L., Robinson J.L., Locati E.H., Ackerman M.J., Benhorin J. (2008). Risk factors for aborted cardiac arrest and sudden cardiac death in children with the congenital long-QT syndrome. Circulation.

[12] Galić E., Bešlić P., Kilić P., Planinić Z., Pašalić A., Galić I., Ćubela V.-V., Pekić P. (2021). Congenital long QT syndrome: A systematic review. Acta Clin. Croat..

[13] Lankaputhra M., Voskoboinik A. (2021). Congenital long QT syndrome: A clinician’s guide. Intern. Med. J..

[14] Postema P.G., Wilde A.A.M. (2014). The measurement of the QT interval. Curr. Cardiol. Rev..

[15] Mittal S.R. (2019). QT interval–its measurement and clinical significance. J. Clin. Prev. Cardiol..

[16] Khatib R., Sabir F.R., Omari C., Pepper C., Tayebjee M.H. (2021). Managing drug-induced QT prolongation in clinical practice. Postgrad. Med. J..

[17] Zeppenfeld K., Tfelt-Hansen J., De Riva M., Winkel B.G., Behr E.R., Blom N.A., Charron P., Corrado D., Dagres N., De Chillou C. (2022). 2022 ESC Guidelines for the management of patients with ventricular arrhythmias and the prevention of sudden cardiac death: Developed by the task force for the management of patients with ventricular arrhythmias and the prevention of sudden cardiac death of the European Society of Cardiology (ESC) Endorsed by the Association for European Paediatric and Congenital Cardiology (AEPC). Eur. Heart J..

[18] Lane C.M., Bos J.M., Rohatgi R.K., Ackerman M.J. (2018). Beyond the length and look of repolarization: Defining the non-QTc electrocardiographic profiles of patients with congenital long QT syndrome. Heart Rhythm.

[19] Schwartz P.J., Priori S.G., Spazzolini C., Moss A.J., Vincent G.M., Napolitano C., Denjoy I., Guicheney P., Breithardt G.N., Keating M.T. (2001). Genotype-phenotype correlation in the long-QT syndrome: Gene-specific triggers for life-threatening arrhythmias. Circulation.

[20] Garson A., Dick M., Fournier A., Gillette P.C., Hamilton R., Kugler J.D., Van Hare G., Vetter V., Vick G. (1993). The long QT syndrome in children. An international study of 287 patients. Circulation.

[21] Passman R., Kadish A. (2001). Polymorphic ventricular tachycardia, long QT syndrome, and torsades de pointes. Med. Clin. N. Am..

[22] Ackerman M.J., Priori S.G., Willems S., Berul C., Brugada R., Calkins H., Camm A.J., Ellinor P.T., Gollob M., Hamilton R. (2011). HRS/EHRA expert consensus statement on the state of genetic testing for the channelopathies and cardiomyopathies: This document was developed as a partnership between the Heart Rhythm Society (HRS) and the European Heart Rhythm Association (EHRA). Europace.

[23] Katritsis D.G., Siontis G.C., Camm A.J. (2013). Prognostic significance of ambulatory ECG monitoring for ventricular arrhythmias. Progress. Cardiovasc. Dis..

[24] Priori S.G., Wilde A.A., Horie M., Cho Y., Behr E.R., Berul C., Blom N., Brugada J., Chiang C.-E., Huikuri H. (2013). Executive summary: HRS/EHRA/APHRS expert consensus statement on the diagnosis and management of patients with inherited primary arrhythmia syndromes. Europace.

[25] Schwartz P.J., Moss A.J., Vincent G.M., Crampton R.S. (1993). Diagnostic criteria for the long QT syndrome. An update. Circulation.

[26] Pappone C., Ciconte G., Anastasia L., Gaita F., Grant E., Micaglio E., Locati E.T., Calovic Z., Vicedomini G., Santinelli V. (2023). Right ventricular epicardial arrhythmogenic substrate in long-QT syndrome patients at risk of sudden death. Europace.

[27] Pelliccia A., Sharma S., Gati S., Bäck M., Börjesson M., Caselli S., Collet J.-P., Corrado D., Drezner J.A., Halle M. (2021). 2020 ESC Guidelines on sports cardiology and exercise in patients with cardiovascular disease: The Task Force on sports cardiology and exercise in patients with cardiovascular disease of the European Society of Cardiology (ESC). Eur. Heart J..

[28] Ingles J., Yeates L., Hunt L., McGaughran J., Scuffham P.A., Atherton J., Semsarian C. (2013). Health status of cardiac genetic disease patients and their at-risk relatives. Int. J. Cardiol..

[29] Priori S.G., Napolitano C. (2004). Genetics of cardiac arrhythmias and sudden cardiac death. Ann. N. Y. Acad. Sci..

[30] Schwartz P.J., Spazzolini C., Crotti L., Bathen J., Amlie J.P., Timothy K., Shkolnikova M., Berul C.I., Bitner-Glindzicz M., Toivonen L. (2006). The Jervell and Lange-Nielsen syndrome: Natural history, molecular basis, and clinical outcome. Circulation.

[31] Zareba W., Moss A.J., Schwartz P.J., Vincent G.M., Robinson J.L., Priori S.G., Benhorin J., Locati E.H., Towbin J.A., Keating M.T. (1998). Influence of the genotype on the clinical course of the long-QT syndrome. N. Engl. J. Med..

[32] Priori S.G., Blomström-Lundqvist C., Mazzanti A., Blom N., Borggrefe M., Camm J., Elliott P.M., Fitzsimons D., Hatala R., Authors/Task Force Members (2015). 2015 ESC Guidelines for the management of patients with ventricular arrhythmias and the prevention of sudden cardiac death: The Task Force for the Management of Patients with Ventricular Arrhythmias and the Prevention of Sudden Cardiac Death of the European Society of Cardiology (ESC) Endorsed by: Association for European Paediatric and Congenital Cardiology (AEPC). EP Eur..

[33] Goldenberg I., Zareba W., Moss A.J. (2008). Long QT syndrome. Curr. Probl. Cardiol..

[34] Früh A., Siem G., Holmström H., Døhlen G., Haugaa K.H. (2016). The Jervell and Lange-Nielsen syndrome; atrial pacing combined with ß-blocker therapy, a favorable approach in young high-risk patients with long QT syndrome?. Heart Rhythm.

[35] Green J.D., Schuh M.J., Maddern B.R., Haymond J., Helffrich R.A. (2000). Cochlear implantation in Jervell and Lange-Nielsen syndrome. Ann. Otol. Rhinol. Laryngol..

[36] Ackerman M.J., Priori S.G., Dubin A.M., Kowey P., Linker N.J., Slotwiner D., Triedman J., Van Hare G.F., Gold M.R. (2016). Beta-blocker therapy for long QT syndrome and catecholaminergic polymorphic ventricular tachycardia: Are all beta-blockers equivalent?. Heart Rhythm.

[37] Nakano Y., Shimizu W. (2016). Genetics of long-QT syndrome. J. Hum. Genet..

[38] Priori S.G., Schwartz P.J., Napolitano C., Bloise R., Ronchetti E., Grillo M., Vicentini A., Spazzolini C., Nastoli J., Bottelli G. (2003). Risk stratification in the long-QT syndrome. N. Engl. J. Med..

[39] Mizusawa Y., Horie M., Wilde A.A. (2014). Genetic and clinical advances in congenital long QT syndrome. Circ. J..

[40] Yap Y.G., Camm A.J. (2003). Drug induced QT prolongation and torsades de pointes. Heart.

[41] Klein R., Ganelin R., Marks J., Usher P., Richards C. (1963). Periodic paralysis with cardiac arrhythmia. J. Pediatr..

[42] Wójcik-Borowska K., Chrościńska-Krawczyk M., Zienkiewicz E., Małka M., Klatka M. (2018). Andersen-Tawil Syndrome—A Case Report. Neurol. Dziecięca.

[43] Onore M.E., Picillo E., D’Ambrosio P., Morra S., Nigro V., Politano L. (2024). Phenotypic Variability of Andersen–Tawil Syndrome Due to Allelic Mutation c. 652C> T in the KCNJ2 Gene—A New Family Case Report. Biomolecules.

[44] Andersen E.D., Krasilnikoff P.A., Overvad H. (1971). Intermittent muscular weakness, extrasystoles, and multiple developmental anomalies: A new syndrome?. Acta Paediatr..

[45] Tawil R., Ptacek L.J., Pavlakis S.G., DeVivo D.C., Penn A.S., Özdemir C., Griggs R.C. (1994). Andersen’s syndrome: Potassium-sensitive periodic paralysis, ventricular ectopy, and dysmorphic features. Ann. Neurol..

[46] Donaldson M., Yoon G., Fu Y.H., Ptacek L. (2004). Andersen-Tawil syndrome: A model of clinical variability, pleiotropy, and genetic heterogeneity. Ann. Med..

[47] Santana L.F., Cheng E.P., Lederer W.J. (2010). How does the shape of the cardiac action potential control calcium signaling and contraction in the heart?. J. Mol. Cell Cardiol..

[48] Barrón-Díaz D.R., Totomoch-Serra A., Escobar-Cedillo R.E., García-Gutierrez A., Reyes-Quintero Á.E., Villegas Davirán S.E., Ibarra-Miramón C.B., Márquez M.F. (2021). Andersen-Tawil syndrome with high risk of sudden cardiac death in four mexican patients. Cardiac and extra-cardiac phenotypes. Rev. Investig. Clin..

[49] Nguyen H.-L., Pieper G.H., Wilders R. (2013). Andersen–Tawil syndrome: Clinical and molecular aspects. Int. J. Cardiol..

[50] Tristani-Firouzi M., Etheridge S.P. (2010). Kir 2.1 channelopathies: The Andersen–Tawil syndrome. Pflugers Arch..

[51] Yoon G., Quitania L., Kramer J., Fu Y., Miller B., Ptacek L. (2006). Andersen–Tawil syndrome: Definition of a neurocognitive phenotype. Neurology.

[52] Tan S.V., Z’graggen W.J., BoËrio D., Rayan D.L.R., Howard R., Hanna M.G., Bostock H. (2012). Membrane dysfunction in Andersen-Tawil syndrome assessed by velocity recovery cycles. Muscle Nerve.

[53] Kukla P., K Biernacka E., Baranchuk A., Jastrzebski M., Jagodzinska M. (2014). Electrocardiogram in Andersen-Tawil syndrome. New electrocardiographic criteria for diagnosis of type-1 Andersen-Tawil syndrome. Curr. Cardiol. Rev..

[54] Vivekanandam V., Männikkö R., Skorupinska I., Germain L., Gray B., Wedderburn S., Kozyra D., Sud R., James N., Holmes S. (2022). Andersen–Tawil syndrome: Deep phenotyping reveals significant cardiac and neuromuscular morbidity. Brain.

[55] Statland J.M., Fontaine B., Hanna M.G., Johnson N.E., Kissel J.T., Sansone V.A., Shieh P.B., Tawil R.N., Trivedi J., Cannon S.C. (2018). Review of the diagnosis and treatment of periodic paralysis. Muscle Nerve.

[56] Dufendach K.A., Timothy K., Ackerman M.J., Blevins B., Pflaumer A., Etheridge S., Perry J., Blom N.A., Temple J., Chowdhury D. (2018). Clinical outcomes and modes of death in Timothy syndrome: A multicenter international study of a rare disorder. JACC Clin. Electrophysiol..

[57] Reichenbach H., Meister E., Theile H. (1992). The heart-hand syndrome. A new variant of disorders of heart conduction and syndactylia including osseous changes in hands and feet. Kinderarztl. Prax..

[58] Splawski I., Timothy K.W., Sharpe L.M., Decher N., Kumar P., Bloise R., Napolitano C., Schwartz P.J., Joseph R.M., Condouris K. (2004). CaV1.2 calcium channel dysfunction causes a multisystem disorder including arrhythmia and autism. Cell.

[59] Krause U., Gravenhorst V., Kriebel T., Ruschewski W., Paul T. (2011). A rare association of long QT syndrome and syndactyly: Timothy syndrome (LQT 8). Clin. Res. Cardiol..

[60] Borbás J., Vámos M., Hategan L., Hanák L., Farkas N., Szakács Z., Csupor D., Tél B., Kupó P., Csányi B. (2022). Geno-and phenotypic characteristics and clinical outcomes of CACNA1C gene mutation associated Timothy syndrome,“cardiac only” Timothy syndrome and isolated long QT syndrome 8: A systematic review. Front. Cardiovasc. Med..

[61] Diep V., Seaver L.H. (2015). Long QT syndrome with craniofacial, digital, and neurologic features: Is it useful to distinguish between Timothy syndrome types 1 and 2?. Am. J. Med. Genet. A.

[62] Bauer R., Timothy K.W., Golden A. (2021). Update on the molecular genetics of Timothy syndrome. Front. Pediatr..

[63] Walsh M.A., Turner C., Timothy K.W., Seller N., Hares D.L., James A.F., Hancox J.C., Uzun O., Boyce D., Stuart A.G. (2018). A multicentre study of patients with Timothy syndrome. EP Eur..

[64] Hiippala A., Tallila J., Myllykangas S., Koskenvuo J.W., Alastalo T.P. (2015). Expanding the phenotype of Timothy syndrome type 2: An adolescent with ventricular fibrillation but normal development. Am. J. Med. Genet. A.

[65] Napolitano C., Bloise R., Priori S.G. (2014). Timothy syndrome. Cardiac Electrophysiology: From Cell to Bedside.

[66] Algra A., Tijssen J., Roelandt J., Pool J., Lubsen J. (1993). QT interval variables from 24 hour electrocardiography and the two year risk of sudden death. Heart.

[67] Gussak I., Brugada P., Brugada J., Wright R.S., Kopecky S.L., Chaitman B.R., Bjerregaard P. (2001). Idiopathic short QT interval: A new clinical syndrome?. Cardiology.

[68] Hancox J.C., Du C., Butler A., Zhang Y., Dempsey C.E., Harmer S.C., Zhang H. (2023). Pro-arrhythmic effects of gain-of-function potassium channel mutations in the short QT syndrome. Philos. Trans. R. Soc. Lond. B Biol. Sci..

[69] Thorsen K., Dam V.S., Kjaer-Sorensen K., Pedersen L.N., Skeberdis V.A., Jurevičius J., Treinys R., Petersen I.M., Nielsen M.S., Oxvig C. (2017). Loss-of-activity-mutation in the cardiac chloride-bicarbonate exchanger AE3 causes short QT syndrome. Nat. Commun..

[70] Raschwitz L.S., El-Battrawy I., Schlentrich K., Besler J., Veith M., Roterberg G., Liebe V., Schimpf R., Lang S., Wolpert C. (2020). Differences in short QT syndrome subtypes: A systematic literature review and pooled analysis. Front. Genet..

[71] Giustetto C., Di Monte F., Wolpert C., Borggrefe M., Schimpf R., Sbragia P., Leone G., Maury P., Anttonen O., Haissaguerre M. (2006). Short QT syndrome: Clinical findings and diagnostic–therapeutic implications. Eur. Heart J..

[72] Campuzano O., Sarquella-Brugada G., Cesar S., Arbelo E., Brugada J., Brugada R. (2018). Recent advances in short QT syndrome. Front. Cardiovasc. Med..

[73] Dewi I.P., Dharmadjati B.B. (2020). Short QT syndrome: The current evidences of diagnosis and management. J. Arrhythmia.

[74] El-Battrawy I., Schlentrich K., Besler J., Liebe V., Schimpf R., Lang S., Odening K.E., Wolpert C., Zhou X., Borggrefe M. (2020). Sex-differences in short QT syndrome: A systematic literature review and pooled analysis. Eur. J. Prev. Cardiol..

[75] Patel U., Pavri B.B. (2009). Short QT syndrome: A review. Cardiol. Rev..

[76] Bjerregaard P. (2018). Diagnosis and management of short QT syndrome. Heart Rhythm.

[77] Veltmann C., Borggrefe M. (2011). A’Schwartz score’for short QT syndrome. Nat. Rev. Cardiol..

[78] Gaita F., Giustetto C., Bianchi F., Schimpf R., Haissaguerre M., Calò L., Brugada R., Antzelevitch C., Borggrefe M., Wolpert C. (2004). Short QT Syndrome: Pharmacological Treatment. J. Am. Coll. Cardiol..

[79] Wolpert C., Schimpf R., Giustetto C., Antzelevitch C., Cordeiro J., Dumaine R., Brugada R., Hong K., Bauersfeld U., Gaita F. (2005). Further insights into the effect of quinidine in short QT syndrome caused by a mutation in HERG. J. Cardiovasc. Electrophysiol..

[80] Milberg P., Tegelkamp R., Osada N., Schimpf R., Wolpert C., Breithardt G., Borggrefe M., Eckardt L. (2007). Reduction of dispersion of repolarization and prolongation of postrepolarization refractoriness explain the antiarrhythmic effects of quinidine in a model of short QT syndrome. J. Cardiovasc. Electrophysiol..

[81] Schimpf R., Veltmann C., Giustetto C., Gaita F., Borggrefe M., Wolpert C. (2007). In vivo effects of mutant HERG K+ channel inhibition by disopyramide in patients with a short QT-1 syndrome: A pilot study. J. Cardiovasc. Electrophysiol..

[82] Bjerregaard P., Gussak I. (2004). Atrial fibrillation in the setting of familial short QT interval. Heart Rhythm.

[83] Bayliss C.E., Beanlands D.S., Baird R. (1968). The pacemaker-twiddler’s syndrome: A new complication of implantable transvenous pacemakers. Can. Med. Assoc. J..

[84] Gomez J.O., Doukky R., Pietrasik G., Wigant R.R., Mungee S., Baman T.S. (2023). Prevalence and predictors of Twiddler’s syndrome. Pacing Clin. Electrophysiol..

[85] Montisci R., Soro C., Demelas R., Agus E., Follesa A., Siragusa G., Nissardi V. (2024). A case series of the twiddler syndrome. Eur. Heart J. Case Rep..

[86] Dattilo G., Scarano M., Casale M., Sergi M., Quattrocchi S., Parato M., Imbalzano E. (2015). An atypical manifestation of Twiddler syndrome. International J. Cardiol..

[87] Salahuddin M., Cader F.A., Nasrin S., Chowdhury M.Z. (2016). The pacemaker-twiddler’s syndrome: An infrequent cause of pacemaker failure. BMC Res. Notes.

[88] Tahirovic E., Haxhibeqiri-Karabdic I. (2018). Twiddler’s syndrome: Case report and literature review. Heart Views.

[89] Roberts A.E., Allanson J.E., Tartaglia M., Gelb B.D. (2013). Noonan syndrome. Lancet.

[90] Vos E., Leenders E., Werkman S.R., Ten Cate F.E.U., Draaisma J.M. (2022). The added value of the electrocardiogram in Noonan syndrome. Cardiol. Young.

[91] Noonan J.A. (1963). Associated noncardiac malformations in children with congenital heart disease. Midwest. Soc. Pediat Res..

[92] Allanson J.E., Roberts A.E. (2021). Noonan syndrome. Cassidy and Allanson’s Management of Genetic Syndromes.

[93] Tartaglia M., Zampino G., Gelb B. (2010). Noonan syndrome: Clinical aspects and molecular pathogenesis. Mol. Syndromol..

[94] Tajan M., Paccoud R., Branka S., Edouard T., Yart A. (2018). The RASopathy family: Consequences of germline activation of the RAS/MAPK pathway. Endocrine Rev..

[95] Grant A.R., Cushman B.J., Cavé H., Dillon M.W., Gelb B.D., Gripp K.W., Lee J.A., Mason-Suares H., Rauen K.A., Tartaglia M. (2018). Assessing the gene–disease association of 19 genes with the RASopathies using the ClinGen gene curation framework. Hum. Mutat..

[96] Capri Y., Flex E., Krumbach O.H., Carpentieri G., Cecchetti S., Lißewski C., Adariani S.R., Schanze D., Brinkmann J., Piard J. (2019). Activating mutations of RRAS2 are a rare cause of Noonan syndrome. Am. J. Hum. Genet..

[97] Linglart L., Gelb B.D. (2020). Congenital heart defects in Noonan syndrome: Diagnosis, management, and treatment. Am. J. Med. Genet. Part C Semin. Med. Genet..

[98] Sun L., Xie Y.-M., Wang S.-S., Zhang Z.-W. (2022). Cardiovascular abnormalities and gene mutations in children with Noonan syndrome. Front. Genet..

[99] Raaijmakers R., Noordam C., Noonan J., Croonen E., Van Der Burgt C., Draaisma J. (2008). Are ECG abnormalities in Noonan syndrome characteristic for the syndrome?. Eur. J. Pediatr..

[100] Armengol A., Brohet C., Lintermans J., Vliers A. (1987). Left ventricle in Noonan’s syndrome. Electro-vecto-echo and angiocardiographic aspects. Arch. Mal. Coeur Vaiss..

[101] Bertola D.R., Chong A.K., Sugayama S.M., Albano L.M.J., Wagenführ J., Moysés R.L., Gonzalez C.H. (2000). Cardiac findings in 31 patients with Noonan’s syndrome. Arq. Bras. Cardiol..

[102] Ichikawa Y., Kuroda H., Ikegawa T., Kawai S., Ono S., Kim K.-S., Yanagi S., Kurosawa K., Aoki Y., Iwamoto M. (2023). Electrocardiographic Changes with Age in Japanese Patients with Noonan Syndrome. J. Cardiovasc. Dev. Dis..

[103] Zenker M., Edouard T., Blair J.C., Cappa M. (2022). Noonan syndrome: Improving recognition and diagnosis. Arch. Dis. Child..

[104] Van der Burgt I. (2007). Noonan syndrome. Orphanet J. Rare Dis..

[105] Vergara P., Altizio S., Falasconi G., Pannone L., Gulletta S., Della Bella P. (2021). Electrophysiological substrate in patients with Barlow’s disease. Arrhythm. Electrophysiol. Rev..

[106] Cheng T.O. (2014). John B. Barlow: The man and his syndrome. Int. J. Cardiol..

[107] Barlow J. (1965). Conjoint clinic on the clinical significance of late systolic murmurs and non-ejection systolic clicks. J. Chronic Dis..

[108] Tamura K., Fukuda Y., Ishizaki M., Masuda Y., Yamanaka N., Ferrans V.J. (1995). Abnormalities in elastic fibers and other connective-tissue components of floppy mitral valve. Am. Heart J..

[109] Rabkin E., Aikawa M., Stone J.R., Fukumoto Y., Libby P., Schoen F.J. (2001). Activated interstitial myofibroblasts express catabolic enzymes and mediate matrix remodeling in myxomatous heart valves. Circulation.

[110] Basso C., Perazzolo Marra M., Rizzo S., De Lazzari M., Giorgi B., Cipriani A., Frigo A.C., Rigato I., Migliore F., Pilichou K. (2015). Arrhythmic mitral valve prolapse and sudden cardiac death. Circulation.

[111] Kubala M., Essayagh B., Michelena H.I., Enriquez-Sarano M., Tribouilloy C. (2023). Arrhythmic mitral valve prolapse in 2023: Evidence-based update. Front. Cardiovasc. Med..

[112] Lab M. (1978). Mechanically dependent changes in action potentials recorded from the intact frog ventricle. Circ. Res.

[113] Korovesis T.G., Koutrolou-Sotiropoulou P., Katritsis D.G. (2022). Arrhythmogenic mitral valve prolapse. Arrhythm. Electrophysiol. Rev..

[114] Guy T.S., Hill A.C. (2012). Mitral valve prolapse. Annu. Rev. Med..

[115] Han H.C., Ha F.J., Teh A.W., Calafiore P., Jones E.F., Johns J., Koshy A.N., O’Donnell D., Hare D.L., Farouque O. (2018). Mitral valve prolapse and sudden cardiac death: A systematic review. J. Am. Heart Assoc..

[116] Savage D.D., Levy D., Garrison R.J., Castelli W.P., Kligfield P., Devereux R.B., Anderson S.J., Kannel W.B., Feinleib M. (1983). Mitral valve prolapse in the general population. 3. Dysrhythmias: The Framingham Study. Am. Heart J..

[117] Brunec-Keller M., Scharf C., Radulovic J., Berdat P.A., CH A.J., Vogt P., Duru F., Caselli S. (2022). Barlow disease: Effect of mitral valve repair on ventricular arrhythmias in 82 patients in a retrospective long-term study. J. Cardiovasc. Surg..

[118] Essayagh B., Sabbag A., Antoine C., Benfari G., Yang L.-T., Maalouf J., Asirvatham S., Michelena H., Enriquez-Sarano M. (2020). Presentation and outcome of arrhythmic mitral valve prolapse. J. Am. Coll. Cardiol..

[119] Lotan D., Salazar-Mendiguchía J., Mogensen J., Rathore F., Anastasakis A., Kaski J., Garcia-Pavia P., Olivotto I., Charron P., Biagini E. (2020). Clinical profile of cardiac involvement in Danon disease: A multicenter European registry. Circ. Genom. Precis. Med..

[120] Danon M.J., Oh S.J., DiMauro S., Manaligod J.R., Eastwood A., Naidu S., Schliselfeld L.H. (1981). Lysosomal glycogen storage disease with normal acid maltase. Neurology.

[121] Nishino I., Fu J., Tanji K., Yamada T., Shimojo S., Koori T., Mora M., Riggs J.E., Oh S.J., Koga Y. (2000). Primary LAMP-2 deficiency causes X-linked vacuolar cardiomyopathy and myopathy (Danon disease). Nature.

[122] Xu J., Li Z., Liu Y., Zhang X., Niu F., Zheng H., Wang L., Kang L., Wang K., Xu B. (2021). Danon disease: A case report and literature review. Diagn. Pathol..

[123] Zhai Y., Miao J., Peng Y., Wang Y., Dong J., Zhao X. (2023). Clinical features of Danon disease and insights gained from LAMP-2 deficiency models. Trends Cardiovasc. Med..

[124] D’souza R.S., Levandowski C., Slavov D., Graw S.L., Allen L.A., Adler E., Mestroni L., Taylor M.R. (2014). Danon disease: Clinical features, evaluation, and management. Circ. Heart Fail..

[125] Boucek D., Jirikowic J., Taylor M. (2011). Natural history of Danon disease. Genet. Med..

[126] Prall F.R., Drack A., Taylor M., Ku L., Olson J.L., Gregory D., Mestroni L., Mandava N. (2006). Ophthalmic manifestations of Danon disease. Ophthalmology.

[127] Cenacchi G., Papa V., Pegoraro V., Marozzo R., Fanin M., Angelini C. (2020). Danon disease: Review of natural history and recent advances. Neuropathol. Appl. Neurobiol..

[128] Cheng Z., Fang Q. (2012). Danon disease: Focusing on heart. J. Hum. Genet..

[129] Li X., Li J., Lu M. (2024). Danon disease manifesting as dilated cardiomyopathy in a 37-year-old woman. Eur. Heart J. Cardiovasc. Imaging.

[130] Hong K.N., Eshraghian E.A., Arad M., Argirò A., Brambatti M., Bui Q., Caspi O., de Frutos F., Greenberg B., Ho C.Y. (2023). International consensus on differential diagnosis and management of patients with Danon disease: JACC state-of-the-art review. J. Am. Coll. Cardiol..

[131] Lee T.H., Salomon D.R., Rayment C.M., Antman E.M. (1986). Hypotension and sinus arrest with exercise-induced hyperkalemia and combined verapamil/propranolol therapy. Am. J. Med..

[132] Lizyness K., Dewald O. (2023). BRASH syndrome. StatPearls [Internet].

[133] Shah P., Gozun M., Keitoku K., Kimura N., Yeo J., Czech T., Nishimura Y. (2022). Clinical characteristics of BRASH syndrome: Systematic scoping review. Eur. J. Int. Med..

[134] Majeed H., Khan U., Khan A.M., Khalid S.N., Farook S., Gangu K., Sagheer S., Sheikh A.B. (2023). BRASH syndrome: A systematic review of reported cases. Curr. Probl. Cardiol..

[135] Farkas J.D., Long B., Koyfman A., Menson K. (2020). BRASH syndrome: Bradycardia, renal failure, AV blockade, shock, and hyperkalemia. J. Emerg. Med..

[136] Srivastava S., Kemnic T., Hildebrandt K.R. (2020). BRASH syndrome. BMJ Case Rep. CP.

[137] Ceasovschih A., Șorodoc V., Covantsev S., Balta A., Uzokov J., Kaiser S.E., Almaghraby A., Lionte C., Stătescu C., Sascău R.A. (2024). Electrocardiogram features in non-cardiac diseases: From mechanisms to practical aspects. J. Multidiscip. Healthc..

[138] Bundgaard H., Jøns C., Lodder E.M., Izarzugaza J.M., Romero Herrera J.A., Pehrson S., Tfelt-Hansen J., Ahlberg G., Olesen M.S., Holst A.G. (2018). A novel familial cardiac arrhythmia syndrome with widespread ST-segment depression. N. Engl. J. Med..

[139] Christensen A.H., Vissing C.R., Pietersen A., Tfelt-Hansen J., Hartvig Lindkær Jensen T., Pehrson S., Henriksen F.L., Sandgaard N.C.F., Iversen K.K., Jensen H.K. (2022). Electrocardiographic findings, arrhythmias, and left ventricular involvement in familial ST-depression syndrome. Circ. Arrhythm. Electrophysiol..

[140] Frosted R., Paludan-Müller C., Vad O.B., Olesen M.S., Bundgaard H., van Dam P., Christensen A.H. (2023). CineECG analysis provides new insights into Familial ST-segment Depression Syndrome. Europace.

[141] Christensen A.H., Nyholm B.C., Vissing C.R., Pietersen A., Tfelt-Hansen J., Olesen M.S., Pehrson S., Iversen K.K., Jensene H.K., Bundgaard H. (2021). Natural history and clinical characteristics of the first 10 Danish families with familial ST-depression syndrome. J. Am. Coll. Cardiol..

[142] Stătescu C., Anghel L., Benchea L.C., Tudurachi B.S., Leonte A., Zăvoi A., Zota I.M., Prisacariu C., Radu R., Șerban I.L. (2023). A Systematic Review on the Risk Modulators of Myocardial Infarction in the "Young"-Implications of Lipoprotein (a). Int. J. Mol. Sci..

[143] Protonotarios N., Tsatsopoulou A., Patsourakos P., Alexopoulos D., Gezerlis P., Simitsis S., Scampardonis G. (1986). Cardiac abnormalities in familial palmoplantar keratosis. Heart.

[144] Protonotarios A., Asimaki A., Basso C., Xylouri Z., Monda E., Protonotarios I., Crisci G., Abrahms D.J., Anastasakis A., Antoniades L. (2025). Naxos Disease and Related Cardio-Cutaneous Syndromes. JACC Adv..

[145] Leopoulou M., Mattsson G., LeQuang J.A., Pergolizzi J.V., Varrassi G., Wallhagen M., Magnusson P. (2020). Naxos disease–a narrative review. Expert. Rev. Cardiovasc. Ther..

[146] Srinivas S.M., Kumar P., Basavaraja G. (2016). Carvajal syndrome. Int. J. Trichol..

[147] Protonotarios I., Asimaki A., Xylouri Z., Protonotarios A., Tsatsopoulou A. (2022). Clinical and molecular aspects of naxos disease. Heart Fail. Clin..

[148] Laxmi R.K., Prithvish C., Sanjay H. (2022). Cardiocutaneous Syndrome: Naxos Disease. Int. J. Preclin. Clin..

[149] Corrado D., Anastasakis A., Basso C., Bauce B., Blomström-Lundqvist C., Bucciarelli-Ducci C., Cipriani A., De Asmundis C., Gandjbakhch E., Jiménez-Jáimez J. (2024). Proposed diagnostic criteria for arrhythmogenic cardiomyopathy: European Task Force consensus report. Int. J. Cardiol..

[150] Finsterer J., Stöllberger C., Wollmann E., Dertinger S., Laccone F. (2016). Autosomal dominant Carvajal plus syndrome due to the novel desmoplakin mutation c. 1678A> T (p. Ile560Phe). Mol. Genet. Metab. Rep..

[151] Brandão M., Bariani R., Rigato I., Bauce B. (2023). Desmoplakin cardiomyopathy: Comprehensive review of an increasingly recognized entity. J. Clin. Med..

[152] Carvajal-Huerta L. (1998). Epidermolytic palmoplantar keratoderma with woolly hair and dilated cardiomyopathy. J. Am. Acad. Dermatol..

[153] Sun Q., Wine Lee L., Hall E.K., Choate K.A., Elder R.W. (2021). Hair and skin predict cardiomyopathies: Carvajal and erythrokeratodermia cardiomyopathy syndromes. Pediatr. Derm..

[154] Wang W., Murray B., Tichnell C., Gilotra N.A., Zimmerman S.L., Gasperetti A., Scheel P., Tandri H., Calkins H., James C.A. (2022). Clinical characteristics and risk stratification of desmoplakin cardiomyopathy. Ep Eur..

[155] Garrod D., Chidgey M. (2008). Desmosome structure, composition and function. Biochim. Biophys. Acta.

[156] Kaplan S.R., Gard J.J., Carvajal-Huerta L., Ruiz-Cabezas J.C., Thiene G., Saffitz J.E. (2004). Structural and molecular pathology of the heart in Carvajal syndrome. Cardiovasc. Pathol..

[157] Di Lorenzo F., Marchionni E., Ferradini V., Latini A., Pezzoli L., Martino A., Romeo F., Iorio A., Bianchi S., Iascone M. (2023). DSP-related cardiomyopathy as a distinct clinical entity? Emerging evidence from an Italian cohort. Int. J. Mol. Sci..

[158] López-Ayala J.M., Gómez-Milanés I., Sánchez Muñoz J.J., Ruiz-Espejo F., Ortíz M., González-Carrillo J., López-Cuenca D., Oliva-Sandoval M., Monserrat L., Valdés M. (2014). Desmoplakin truncations and arrhythmogenic left ventricular cardiomyopathy: Characterizing a phenotype. Europace.

[159] Cipriani A., Bauce B., De Lazzari M., Rigato I., Bariani R., Meneghin S., Pilichou K., Motta R., Aliberti C., Thiene G. (2020). Arrhythmogenic right ventricular cardiomyopathy: Characterization of left ventricular phenotype and differential diagnosis with dilated cardiomyopathy. J. Am. Heart Assoc..

[160] Anghel L., Sascău R., Zota I.M., Stătescu C. (2021). Well-Known and Novel Serum Biomarkers for Risk Stratification of Patients with Non-ischemic Dilated Cardiomyopathy. Int. J. Mol. Sci..

[161] Smith E.D., Lakdawala N.K., Papoutsidakis N., Aubert G., Mazzanti A., McCanta A.C., Agarwal P.P., Arscott P., Dellefave-Castillo L.M., Vorovich E.E. (2020). Desmoplakin cardiomyopathy, a fibrotic and inflammatory form of cardiomyopathy distinct from typical dilated or arrhythmogenic right ventricular cardiomyopathy. Circulation.

[162] Augusto J.B., Eiros R., Nakou E., Moura-Ferreira S., Treibel T.A., Captur G., Akhtar M.M., Protonotarios A., Gossios T.D., Savvatis K. (2020). Dilated cardiomyopathy and arrhythmogenic left ventricular cardiomyopathy: A comprehensive genotype-imaging phenotype study. Eur. Heart J. Cardiovasc. Imaging.

[163] Ghawanmeh M., Simon Frances B., Kerai A., Patel P., Du J., Kumar P. (2022). Management of recurrent myocarditis due to desmoplakin cardiomyopathy: Diagnostic and therapeutic challenges. Case Rep..

[164] Reza N., de Feria A., Chowns J.L., Hoffman-Andrews L., Vann L., Kim J., Marzolf A., Owens A.T. (2022). Cardiovascular characteristics of patients with genetic variation in Desmoplakin (DSP). Cardiogenetics.

[165] Jacobsen A.P., Chiampas K., Muller S.A., Gasperetti A., Yanek L.R., Carrick R.T., Gordon C., Tichnell C., Murray B., Calkins H. (2024). Endurance exercise promotes episodes of myocardial injury in individuals with a pathogenic desmoplakin (DSP) variant. Heart Rhythm.

[166] Lemus Barrios G.A., Lopez-Lopez J.P., Barbosa-Balaguera S., Correa A.M. (2023). Left-dominant arrhythmogenic cardiomyopathy due to desmoplakin mutation: A case report. ESC Heart Fail..

